# Ocean Carbon Dioxide Removal and Storage

**DOI:** 10.1021/acs.chemrev.5c00433

**Published:** 2026-01-06

**Authors:** Chang-Ho Lee, Adam V. Subhas, Ju-Hyoung Kim, Kitack Lee

**Affiliations:** † Division of Environmental Science and Engineering, 34995 Pohang University of Science and Technology, Pohang 790-784, Korea; ‡ Department of Marine Chemistry and Geochemistry, Woods Hole Oceanographic Institution, Woods Hole, Massachusetts 02543-1050, United States; § Department of Aquaculture and Aquatic Science, 65450 Kunsan National University, Gunsan 54150, Korea; ∥ Fisheries Science Institute, 65450 Kunsan National University, Gunsan 54150, Korea

## Abstract

The ocean, Earth’s largest carbon reservoir, exerts a central role over atmospheric CO_2_ through its capacity to store carbon primarily as bicarbonate ions. Direct observations indicate that the global ocean has a net carbon uptake of 2.6–3.0 petagrams of carbon annually, representing nearly 30% of anthropogenic CO_2_ emissions. This review examines two principal domains of oceanic carbon cycling. The first concerns the natural uptake and storage of anthropogenic CO_2_, with emphasis on the response of the marine carbonate system and the spatial distribution of absorbed carbon. The second addresses emerging marine CO_2_ removal strategies, especially ocean alkalinity enhancement and macroalgae-based approaches. Ocean alkalinity enhancement aims to increase seawater buffering capacity to facilitate greater CO_2_ uptake, whereas macroalgae-based strategies rely on photosynthetic fixation and the subsequent storage of organic and inorganic carbon in various reservoirs. Effective implementation of these approaches necessitates rigorous monitoring, reporting, and verification frameworks to ensure their quantifiable efficacy and environmental integrity.

## Graphical abstract

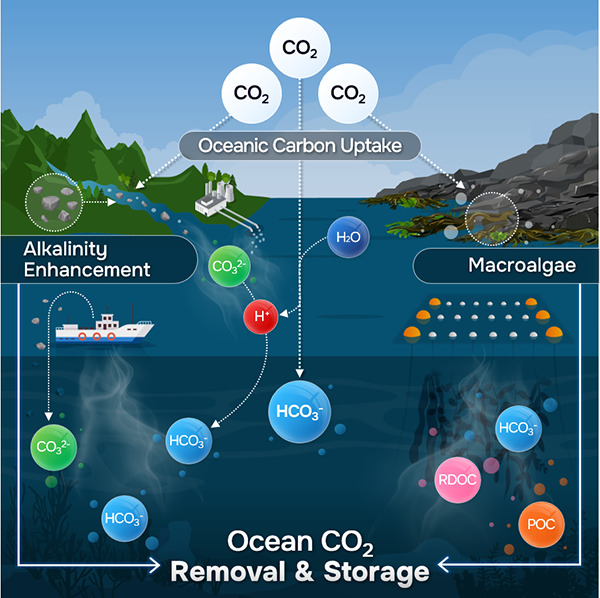

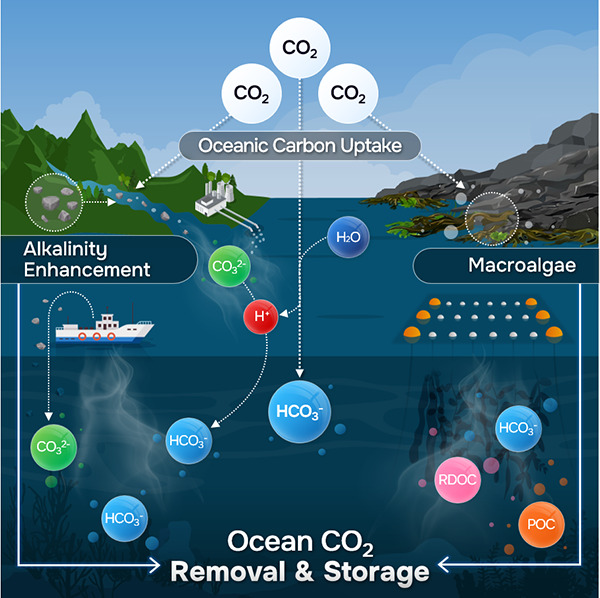

## 1 Introduction

Since the Industrial Revolution, human activities—primarily fossil fuel combustion, land-use change, and cement production—have elevated atmospheric carbon dioxide (CO_2_) concentrations to more than 420 ppm by 2024,^1,2^ an increase unprecedented. This rapid rise constitutes the dominant driver of contemporary climate change, perturbing the Earth’s radiative energy balance and intensifying its greenhouse effect.^3–5^ Although the climatic consequence of rising CO_2_ was recognized more than a century ago,^6^ current emission trajectories indicate continued growth in the absence of stringent mitigation.^2^

The ocean exerts a critical moderating influence on this rise (Figure 1), absorbing approximately 30% of anthropogenic CO_2_ emissions.^7,8^ Through this uptake, it slows atmospheric accumulation and thereby mitigates the rate of global warming.^9^ For millennia prior to industrialization, the global carbon cycle was near equilibrium, with atmospheric CO_2_ stabilized at near 280 ppm.^10^ Anthropogenic emissions have disrupted this balance, enhancing ocean uptake, altering subsurface pathways, and modifying seawater–carbonate chemistry.^11–13^ However, the buffering capacity of the ocean is not infinite. Ongoing climate-induced changes in circulation, stratification, and biological productivity are projected to further limit the efficiency and durability of marine CO_2_ storage.^13–15^

**Figure 1:**
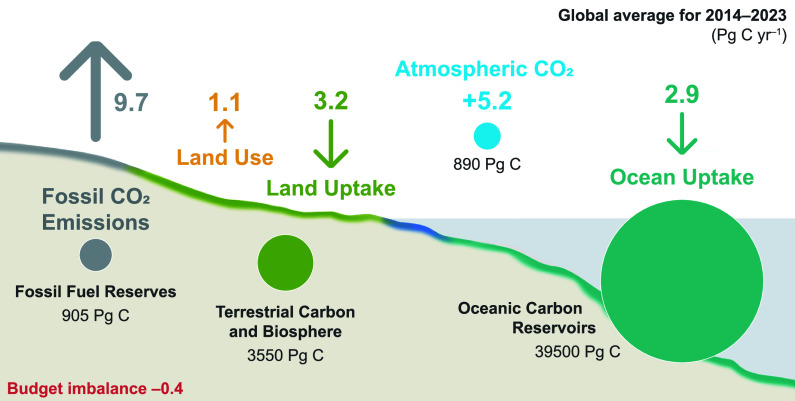
Schematic of the global carbon cycle illustrating anthropogenic CO_2_ fluxes for 2014–2023. During this period, the land and ocean each absorbed approximately 30% of anthropogenic CO_2_ emissions, while the remaining ∼ 40% accumulated in the atmosphere, driving the continued increase in atmospheric CO_2_ concentrations. Adapted from ref 2. Copyright 2025 Copernicus Publications under Creative Commons CC BY license https://creativecommons.org/licenses/by/4.0/.

Although natural ocean uptake has moderated the pace of atmospheric CO_2_ increase, it remains insufficient to achieve climate stabilization, which requires additional net removal on the order of gigatonnes of carbon per year.^5,16,17^ This constraint has stimulated interest in marine carbon dioxide removal (mCDR)—engineered interventions aimed at enhancing the ocean’s carbon storage potential.^17^ Despite their conceptual promise, mCDR approaches face substantial uncertainties regarding storage efficiency, permanence, scalability, and potential ecological and social implications.^17^

This review evaluates the chemical principles underlying both natural and engineered pathways of oceanic carbon uptake. Section 2 outlines the thermodynamic framework of the seawater–carbonate system and Section 3 describes the mechanisms governing CO_2_ absorption and its redistribution into the ocean interior. Together, these sections establish the natural context for ocean carbon storage and clarify its sensitivity to anthropogenic perturbations. Building on this foundation, Sections 4 and 5 address two biogeochemically grounded and potentially scalable mCDR approaches: ocean alkalinity enhancement and macroalgae-based strategies. Ocean alkalinity enhancement strengthens the carbonate buffer by introducing alkaline minerals, thereby increasing CO_2_ uptake capacity and extending storage time scales, whereas macroalgae-based approaches rely on photosynthetic fixation followed by the retention of organic or inorganic carbon in long-lived reservoirs. Presenting these approaches through the unifying chemistry of the carbonate system provides general chemists with a coherent framework for understanding how engineered interventions leverage natural ocean processes to enhance carbon removal.

## 2 Thermodynamic Responses of the Carbonate System to Anthropogenic CO_2_

### 2.1 Gas Exchange and Hydration Reaction

Atmospheric CO_2_ enters the ocean via air–sea gas exchange and dissolves as aqueous CO_2_ (Figure 2a), governed by Henry’s law:

$${\mathrm{CO}}_{2}\left(gas\right)↔{\mathrm{CO}}_{2}\left(aq\right),K_{0}=\left[{\mathrm{CO}}_{2}\right]/f{\mathrm{CO}}_{2}$$ 1

**Figure 2:**
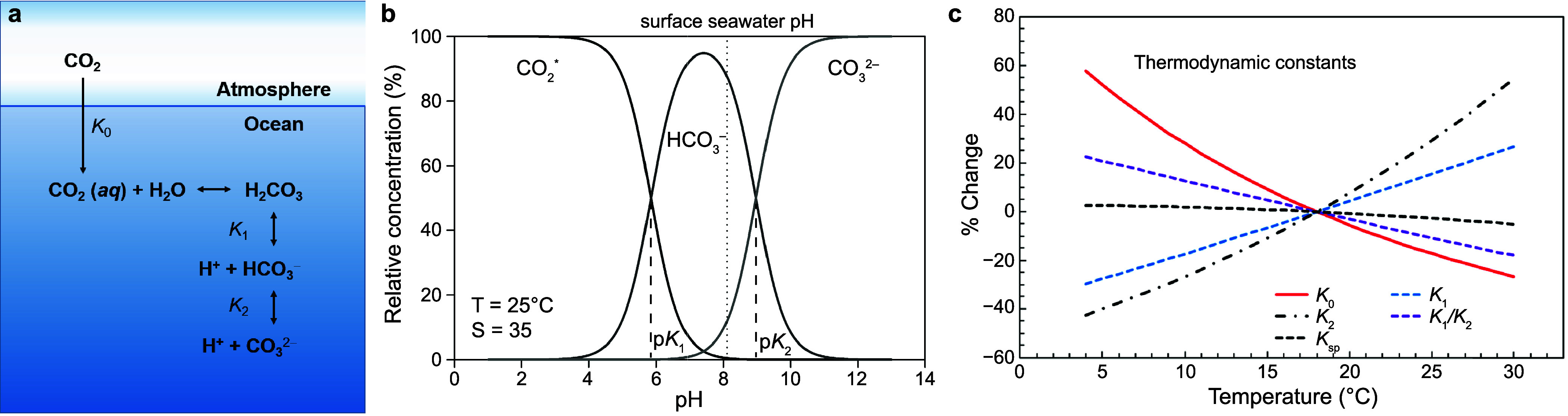
(**a**) Equilibration of CO_2_ in seawater. (**b**) Bjerrum plot showing the relative concentrations of dissolved inorganic carbon species (CO_2_*, HCO_3_^–^, and CO_3_^2–^) as a function of pH. Species distributions were calculated using the carbonic acid dissociation constants from ref 24 as refitted by ref 25, under conditions of 25 °C and salinity 35. (**c**) Variation in the thermodynamic constants governing the carbonate system. *K*_0_ denotes the solubility constant, *K*_1_ and *K*_2_ represent the first and second dissociation constants of carbonic acid. Reproduced from ref 26. Copyright 2020 Springer Nature under Creative Commons CC BY license https://creativecommons.org/licenses/by/4.0/.

Once dissolved, CO_2_ undergoes hydration:

$${\mathrm{CO}}_{2}\left(aq\right)+H_{2}O↔H_{2}{\mathrm{CO}}_{3}$$ 2

Under typical oceanic pH, this reaction is kinetically limited. The hydration rate is ∼ 0.03 s^–1^, meaning only 3% of dissolved CO_2_ hydrate per second, while the reverse reaction (dehydration) is ∼ 1,000 times faster.^18,19^ This kinetic asymmetry arises from the high free energy barrier and significant entropic cost of hydration, which involves proton transfer mediated by multiple water molecules.^20,21^ Quantum chemical studies indicate that a minimum of three water molecules is required to form a low-energy transition state, reducing the activation energy.^20^

Even when formed, carbonic acid (H_2_CO_3_) is highly unstable, rapidly dissociating into bicarbonate and proton. Its fleeting intermediate is only marginally stabilized by microsolvation, where hydrogen bonding with surrounding water molecules lowers enthalpy but imposes an entropic penalty.^20^ Due to the negligible concentration and transient nature of H_2_CO_3_ in seawater, the sum of free CO_2_(*aq*) and H_2_CO_3_ is commonly treated as a single species—denoted as CO_2_* in carbonate system models.^22,23^

### 2.2 Equilibrium in Seawater

Dissolved CO_2_* in seawater rapidly dissociates into bicarbonate (HCO_3_^–^) and carbonate (CO_3_^2–^), resulting in three principal forms of dissolved inorganic carbon (DIC): CO_2_*, HCO_3_^–^, and CO_3_^2–^ (Figure 2a). Their relative abundances are governed by the first and second dissociation constants (*K*_1_ and *K*_2_) of H_2_CO_3_:

$${\mathrm{CO}}_{2}^{*}+H_{2}O↔H^{+}+{{\mathrm{HCO}}_{3}}^{-},K_{1}=\left[H^{+}\right]\left[{{\mathrm{HCO}}_{3}}^{-}\right]/\left[{\mathrm{CO}}_{2}^{*}\right]$$ 3

$${{\mathrm{HCO}}_{3}}^{-}↔H^{+}+{{\mathrm{CO}}_{3}}^{2-},K_{2}=\left[H^{+}\right]\left[{{\mathrm{CO}}_{3}}^{2-}\right]/\left[{{\mathrm{HCO}}_{3}}^{-}\right]$$ 4

These equilibria establish on time scales of minutes or less. Under surface ocean conditions, ∼ 90% of DIC exists as HCO_3_^–^, ∼ 9% as CO_3_^2–^, and ∼ 1% as CO_2_(*aq*) and undissociated H_2_CO_3_ (Figure 2b).

The equilibrium constants *K*_0_, *K*_1_, and *K*_2_ are primarily temperature (*T*)-dependent (Figure 2c), with secondary sensitivity to salinity (*S*) and pressure (*P*).^22^ Together, these equilibria can be expressed in the overall equation:

$${{\mathrm{CO}}_{2}}^{*}+H_{2}O+{{\mathrm{CO}}_{3}}^{2-}↔2{{\mathrm{HCO}}_{3}}^{-},K=K_{1}/K_{2}$$ 5

This formulation provides a useful framework for predicting shifts in carbon speciation under changing oceanic conditions. For example, increasing *T* reduces CO_2_ solubility (*K*_0_), while increasing *K*_1_ and *K*_2_ at different rates, leading to a decreased *K*_1_/*K*_2_ ratio (Figure 2c). In a closed system, these thermodynamic shifts drive an increase in gaseous and aqueous CO_2_ as well as CO_3_^2–^, accompanied by a decrease in HCO_3_^–^ concentration.^22,26^

### 2.3 Analytical Parameters of the Seawater–Carbonate System

This section describes the properties of the four key carbonate parameters, reviews the traditional analytical methods for discrete samples, and highlights recent developments in autonomous instruments for in situ observation.

#### 2.3.1 Total Dissolved Inorganic Carbon

Total dissolved inorganic carbon is the sum of three species in seawater (DIC = [CO_2_*] + [HCO_3_^–^] + [CO_3_^2–^]). It is measured by acidifying the sample to convert all species to CO_2_ gas, which is then extracted and quantified. The standard technique is coulometric detection, where the released CO_2_ is absorbed into ethanolamine solution (HO­(CH_2_)_2_NH_2_) and titrated electrochemically.^27–31^ Reported in moles per kilogram of solution, DIC is unaffected by *T* and *P*. Because the number of carbon atoms is conserved, its concentration changes conservatively during mixing and can be calculated from end-member values and mixing ratios.^22,32^

#### 2.3.2 Total Alkalinity

Total alkalinity (TA) is a measure of the capacity of seawater to neutralize acids, representing the excess of proton acceptors over proton donors. According to Dickson’s proton-balance definition,^33^ TA is referenced to the H_2_CO_3_ equivalence point in a titration and is expressed as

$$\mathrm{TA}=\left[{{\mathrm{HCO}}_{3}}^{-}\right]+2\left[{{\mathrm{CO}}_{3}}^{2-}\right]+\left[B{{\left(\mathrm{OH}\right)}_{4}}^{-}\right]+\left[{\mathrm{OH}}^{-}\right]+\left[{{\mathrm{HPO}}_{4}}^{2-}\right]+2\left[{{\mathrm{PO}}_{4}}^{3-}\right]+\left[\mathrm{SiO}{{\left(\mathrm{OH}\right)}_{3}}^{-}\right]+\left[{\mathrm{NH}}_{3}\right]+\left[{\mathrm{HS}}^{-}\right]-{\left[H^{+}\right]}_{F}-\left[{{\mathrm{HSO}}_{4}}^{-}\right]-\left[\mathrm{HF}\right]-\left[H_{3}{\mathrm{PO}}_{4}\right]+\left[\mathrm{unknown bases}-\mathrm{unknown acids}\right]$$ 6

where [H^+^]_F_ denotes the concentration of free hydrogen ions and the final term accounts for unidentified acid–base species, including organic acids (see Section 2.4.3). TA is unaffected by CO_2_ exchange or H_2_CO_3_ addition, because its dissociation releases a proton (H^+^) and its conjugate base (HCO_3_^–^) in equivalent amounts. This balance preserves TA, making it a stable property of the seawater–carbonate system.

TA is determined through acidimetric titration,^27,33–36^ adding a known quantity of strong acid until all carbonate species are eventually converted to CO_2_*. Expressed in moles per kilogram of solutions, TA is conservative with respect to *T*, *P*, and mixing^9,22,32^ as demonstrated by its conservative formulation:

$${\mathrm{TA}}_{\mathrm{ec}}=\left[{\mathrm{Na}}^{+}\right]+2\left[{\mathrm{Mg}}^{2+}\right]+2\left[{\mathrm{Ca}}^{2+}\right]+\left[K^{+}\right]+2\left[{\mathrm{Sr}}^{2+}\right]-\left[{\mathrm{Cl}}^{-}\right]-\left[{\mathrm{Br}}^{-}\right]+\cdot \cdot \cdot +{\mathrm{TNH}}_{3}-{\mathrm{TNO}}_{3}-{\mathrm{TNO}}_{2}-{\mathrm{TPO}}_{4}-2{\mathrm{TSO}}_{4}-\mathrm{TF}$$ 7

where TNH_3_ = [NH_3_] + [NH_4_^+^], TNO_3_ = [NO_3_^–^] + [HNO_3_], TNO_2_ = [NO_2_^–^] + [HNO_2_], TPO_4_ = [H_3_PO_4_] + [H_2_PO_4_^–^] + [HPO_4_^2–^] + [PO_4_^3–^], TSO_4_ = [H_2_SO_4_] + [HSO_4_^–^] + [SO_4_^2–^], and TF = [HF] + [F^–^]. Because seawater is electrically neutral, Equation 7 is equivalent to Dickson’s formulation of TA in Equation 6, with all terms expressed conservatively—demonstrating that TA itself is a conservative property.^9,22,32^

#### 2.3.3 Hydrogen Ion Concentration

The hydrogen ion concentration of seawater ([H^+^]) is typically measured spectrophotometrically.^27,37–45^ The standard indicator, *m*-cresol purple, is added to the sample, where its two dissociated forms exhibit distinct absorbance spectra. The absorbance ratio at two wavelengths, combined with the indicator’s dissociation constants corrected for *S* and *T*, yields [H^+^]. The resulting pH is defined as

$$\mathrm{pH}=-\log_{10}\left[H^{+}\right]$$ 8

where [H^+^] is expressed in moles per kilogram of solution. Seawater pH is most often reported on the total scale,^22,39,46–50^ which includes contributions from free hydrogen and bisulfate ions:

$$\left[H^{+}\right]\approx {\left[H^{+}\right]}_{F}+\left[{{\mathrm{HSO}}_{4}}^{-}\right]$$ 9

Notably, seawater pH is sensitive to changes in *T* and *P*, and measurements must account for these factors.

#### 2.3.4 Fugacity of CO_2_

Fugacity of CO_2_ (*f*CO_2_) represents the effective partial pressure or thermodynamic activity of CO_2_ in the gas phase. Unlike partial pressure of CO_2_ (*p*CO_2_), which assumes ideal gas behavior, *f*CO_2_ accounts for the nonideal nature of CO_2_ using fugacity corrections based on *T* and *P*. To determine *f*CO_2_, the mole fraction of CO_2_ in air equilibrated with seawater at a given *T* is measured using an infrared analyzer.^27,37,51–60^ *f*CO_2_ is calculated from the mole fraction of CO_2_ by correcting for water vapor and normalizing the results to a standard atmospheric pressure (1 atm).^23,27,61–63^ The *f*CO_2_ in seawater is highly *T*-dependent, changing by approximately 4.2% per Kelvin.^51–55,60,64,65^ Because the difference between *f*CO_2_ and *p*CO_2_ is less than 1%, the two terms are used interchangeably in this review.

#### 2.3.5 Autonomous Instruments for Measuring Carbonate System Parameters

Advances in autonomous sensor technology complement discrete measurements and ship-based underway systems by enabling high-frequency, long-term in situ observations in remote locations. Among carbonate system parameters, pH and *p*CO_2_ are most developed, with several commercial instruments available for seawater applications.^66,67^ Although the precision and accuracy needed to resolve long-term trends^66,68–72^ remains challenging, autonomous sensors have substantially expanded our capacity to monitor the marine carbonate system across broad spatial and temporal scales.

For pH, two main approaches are widely used: spectrophotometric methods using pH-sensitive indicator dyes (e.g., SAMI-pH, iSAMI, Sunburst Sensors)^70,73,74^ and ion-selective field effect transistor sensors (e.g., SeaFET, Seabird; DuraFET, Honeywell).^75–80^ For *p*CO_2_, the most common approach uses equilibrator-based nondispersive infrared detectors (e.g., CO_2_-Pro, Pro-oceanus; MAPCO_2_, Battelle; C-sense, Turner Designs)^81–83^ or spectrophotometry (e.g., SAMI-CO_2_, Sunburst Sensors).^84–86^ More recently, optode-based *p*CO_2_ sensors using fluorescent dyes have emerged (e.g., Aanderaa 4797, Aanderaa).^68,87,88^ For TA, autonomous instruments employ miniaturized acid–base titration, with pH changes tracked either by spectrophotometry (e.g., SAMI-Alk; Sunburst Sensors)^89,90^ or by ion-selective field effect transistor electrodes.^91^ For DIC, prototype instruments employ either spectrophotometric detection^92,93^ or nondispersive infrared-based quantification^94^ of CO_2_ released after acidification.

### 2.4 Thermodynamic Models for Characterizing the Seawater–Carbonate System

#### 2.4.1 Calculation of Carbonate Species Concentrations from TA and *f*CO_2_

Among the four carbonate system parameters (DIC, TA, pH, and *f*CO_2_), any two can be used to calculate the concentrations of the individual carbonate species ([CO_2_*], [HCO_3_^–^], [CO_3_^2–^]) (Figure 3a). These calculations rely on thermodynamic equilibrium constants, notably *K*_1_ and *K*_2_, as well as constants for other weak acids, all of which vary with *S* and *T* (see Table 1 and corresponding references). In this review, we emphasize the TA–*f*CO_2_ pair, which is particularly useful for estimating anthropogenic CO_2_ in seawater (see Section 3.1).

**Table 1:** Equilibria, Dissociation Constants, and Total Concentrations of Acid–Base Pairs in Seawater^a^

Equilibrium	Constant	Total concentration
CO_2_(*gas*) ↔ CO_2_*(*aq*)	*K*_0_ ^61,95^	
CO_2_*(*aq*) + H_2_O ↔ HCO_3_^–^ + H^+^	*K*_1_ ^24,25,64,96–106^	DIC = [CO_2_*(*aq*)] + [HCO_3_^–^] + [CO_3_^2–^]
HCO_3_^–^ ↔ CO_3_^2–^ + H^+^	*K*_2_ ^24,25,64,96–106^	
B(OH)_3_ + H_2_O ↔ B(OH)_4_^–^ + H^+^	*K*_B_ ^96,107,108^	TB = [B(OH)_3_] + [B(OH)_4_^–^] ^109,110^
H_2_O ↔ OH^–^ + H^+^	*K*_W_ ^111–113^	
H_3_PO_4_ ↔ H_2_PO_4_^–^ + H^+^	*K*_P1_ ^114,115^	TPO_4_ = [H_3_PO_4_] + [H_2_PO_4_^–^] + [HPO_4_^2–^] + [PO_4_^3–^]
H_2_PO_4_^–^ ↔ HPO_4_^2–^ + H^+^	*K*_P2_ ^114,115^	
HPO_4_^2–^ ↔ PO_4_^3–^ + H^+^	*K*_P3_ ^114,115^	
Si(OH)_4_ ↔ SiO(OH)_3_^–^ + H^+^	*K*_Si_ ^115^	TSi = [Si(OH)_4_] + [SiO(OH)_3_^–^]
NH_4_^+^ ↔ NH_3_ + H^+^	*K*_NH4_ ^115,116^	TNH_3_ = [NH_4_^+^] + [NH_3_]
H_2_S ↔ HS^–^ + H^+^	*K*_S1_ ^117^	THS = [H_2_S] + [HS^–^]
HSO_4_^–^ ↔ SO_4_^2–^ + H^+^	*K*_HSO4_ ^47,105,118^	TSO_4_ = [HSO_4_^–^] + [SO_4_^2–^] ^119,120^
HF ↔ F^–^ + H^+^	*K*_F_ ^111,121^	TF = [HF] + [F^–^][Bibr ref122]

a All dissolved species are in the aqueous phase; the (*aq*) notation is omitted for clarity.

**Figure 3:**
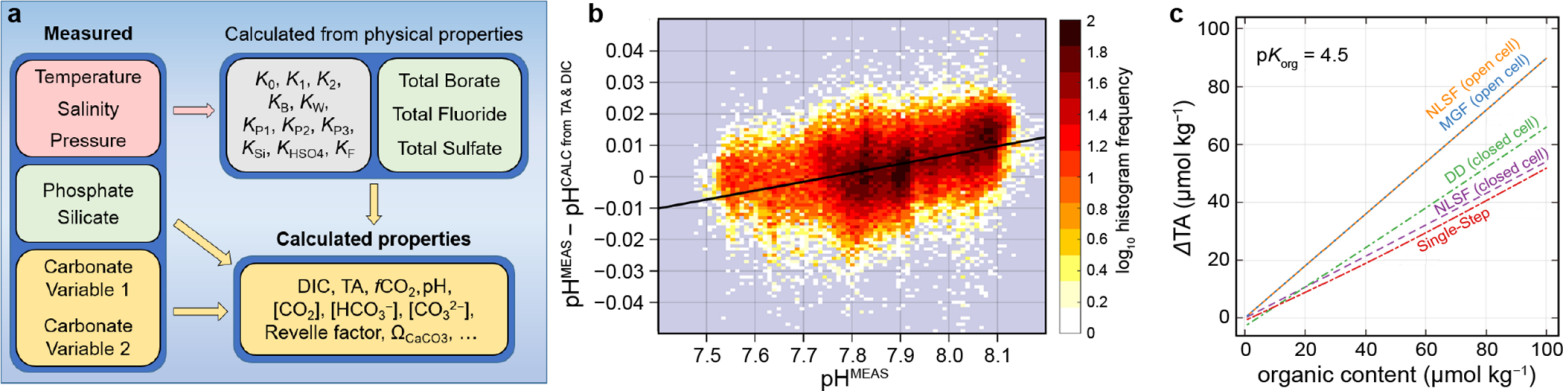
(**a**) Schematic representation of a thermodynamic model that uses measured quantities to calculate carbonate parameters. (**b**) Dependence of pH deviations (ΔpH = pH^MEAS^ – pH^CALC^) on the measured pH, shown as a two-dimensional histogram for measurement frequency. (**c**) Modeled TA deviations (ΔTA = TA^MEAS^ – TA^CALC^) as a function of total organic content. Each line indicates distinct results from different evaluations (NLSF for nonlinear least-squares fitting; MGF for modified Gran function; DD for difference derivative) using titration data (closed or open cell).^123^ (**a**, **b**) Adapted from ref 124. Copyright 2023 John Wiley and Sons under Creative Commons CC BY license https://creativecommons.org/licenses/by/4.0/. (**c**) Adapted with permission from ref 123. Copyright 2020 Elsevier.

The following relationships describe the carbonate species as functions of *f*CO_2_ and [H^+^]:

$$\left[{{\mathrm{CO}}_{2}}^{*}\right]=K_{0}f{\mathrm{CO}}_{2}$$ 10

$$\left[{{\mathrm{HCO}}_{3}}^{-}\right]=K_{0}K_{1}f{\mathrm{CO}}_{2}/\left[H^{+}\right]$$ 11

$$\left[{{\mathrm{CO}}_{3}}^{2-}\right]=K_{0}K_{1}K_{2}f{\mathrm{CO}}_{2}/{\left[H^{+}\right]}^{2}$$ 12

By substituting the expressions for carbonate species into the TA definition (eq 6) and expressing other contributors in terms of their respective dissociation constants and [H^+^],^27^ TA can be reformulated as follows:

$$\mathrm{TA}=K_{0}K_{1}f{\mathrm{CO}}_{2}/\left[H^{+}\right]+2K_{0}K_{1}K_{2}f{\mathrm{CO}}_{2}/{\left[H^{+}\right]}^{2}+\left(K_{B}\mathrm{TB}\right)/\left(\left[H^{+}\right]+K_{B}\right)+K_{W}/\left[H^{+}\right]-\left[H^{+}\right]$$ 13

where each term represents major contributions to TA from bicarbonate, carbonate, borate, hydroxide, and hydrogen ions, respectively; minor species such as phosphate, silicate, ammonia, and organic bases were omitted for clarity. Equation 13 is solved numerically (e.g., Newton–Raphson method) to find [H^+^], which is then used in Equations 10–12 to calculate carbonate species concentrations. The required total concentrations and dissociation constants are summarized in Table 1.

Estimating the borate contribution ([B­(OH)_4_^–^]), up to 5% of TA in surface waters,^110,113^ requires both the total boron concentration (TB)^109,110^ and the dissociation constant of boric acid (*K*_B_).^96,107,108^ The hydroxide contribution ([OH^–^]) is computed from the dissociation constant of water (*K*_W_).^111–113^ Other minor inorganic acid–base species, represented in Equation 6,^33^ account for less than 0.2% of TA in the open ocean but can be significant in coastal waters. Nutrient contributions including phosphate, silicate, and ammonia are typically corrected based on direct measurements from standard colorimetric methods.^125–128^

#### 2.4.2 Computational Packages for Carbonate System Calculations

A variety of computational packages have been developed to facilitate carbonate system calculations. The most widely used are CO2SYS family of programs, originally released for DOS^129^ and later adapted for Microsoft Excel,^130,131^ MATLAB,^132^ Python,^133^ and Julia.^134^ Additional implementations include AquaEnv^135^ and seacarb^136^ in R, swco2 in Excel and Visual Basic,^137,138^ and mocsy in Fortran.^139^ These programs compute unmeasured parameters (e.g., DIC, TA, pH, *f*CO_2_) from a well-constrained input pair and auxiliary data (*T*, *S*, *P*, and nutrients).^140,141^ They also provide calcium carbonate saturation states and sensitivity factor (e.g., the Revelle factor), critical for assessing system responses to perturbations.

Accurate thermodynamic calculations require careful attention of pH scale and equilibrium constants. For most oceanographic applications, pH is reported on the total scale (eq 9), with conversions available from the seawater scale (pH_SW_ = [H^+^]_F_ + [HSO_4_^–^] + [HF]).^23,27,99,103,113,140,141^ The carbonic acid dissociation constants from Mehrbach et al.,^24^ refit by Dickson and Millero,^25^ are widely recommended for typical oceanic systems.^101,102,142–144^ More recent formulations by Lueker et al.^106^ and Millero^108^ are also commonly applied, offering consistent results over broader ranges of *S* and *T* ranges.^27,140,141,145,146^ For boron (B), a widely accepted B-to-chlorinity ratio of 0.2414 mg kg^–1^ is used,^110^ supported by multiple independent studies.^146–148^ Minor deviations have been observed in regions such as the Baltic^149^ and Arctic,^150,151^ where freshwater dilution alter this relationship.

#### 2.4.3 Challenges in Internal Consistency

Despite major advances in thermodynamic models of the marine carbonate system, internal consistency remains a persistent issue in characterizing seawater–carbonate chemistry.^145,152–157^ In practice, measured values of one parameter (e.g., pH^MEAS^) often differ from those calculated from alternative parameter pairs (e.g., pH^CALC^ from TA + DIC). These deviations (e.g., ΔpH = pH^MEAS^ – pH^CALC^) are not random but typically follow systematic patterns such as a dependency of ΔpH on pH itself (Figure 3b).^124,154,155^

Internal consistency can only be evaluated when measurements are of high metrological quality. Although autonomous sensing technologies have advanced rapidly (Section 2.3.5), their uncertainties remain greater than those of traditional benchtop methods (Sections 2.3.1–2.3.4).^66,67,77,79,80^ Consequently, achieving climate-quality targets for long-term trend detection^69^—± 2 μmol kg^–1^ for TA and DIC, ± 0.003 for pH, and ± 0.5% for *p*CO_2_—still depends on discrete bottle-sample analyses. TA and DIC are routinely referenced to certified reference materials, yielding accuracies better than ± 2 μmol kg^–1^, sufficient to meet these standards.^23^ Nevertheless, pH values calculated from well-calibrated TA and DIC often deviate from measured pH beyond the ± 0.003 target.^141,154,155^ Contributing factors include spectrophotometric issues such as indicator impurities (unpurified vs purified *m*-cresol purple), variations in standard operating procedures, and the absence of reference materials covering the full environmental pH range.^155^ Recent advances, including improved purification of indicator and extended characterizations across *S* and *T* ranges, have begun to reduce these discrepancies.^42,44,45,158^

Even with state-of-the-art measurements, uncertainties in equilibrium constants remain a major challenge. The dissociation constants of carbonic acid (*K*_1_ and *K*_2_) are key sources of inconsistency. Different formulations (e.g., Mehrbach et al.,^24^ refit by Dickson and Millero;^25^ refit by Lueker et al.^106^) yield diverging results, and no single set of constants ensures consistency across all *T*–*S*–*P.* ^141,145,157,159^ These discrepancies become more pronounced in extreme environments such as polar or estuarine waters,^145,159^ emphasizing the need to re-evaluate *K*_1_ and *K*_2_ under broader environmental conditions. Refining these constants remains an essential and active research priority.^157,160^

Another source of uncertainty is the contribution of poorly constrained organic bases and acids (Figure 3c), included only implicitly in Dickson’s definition, Equation 6 as “[unknown bases – unknown acids]”. Dissolved organic matter (DOM) contributes to TA through proton-accepting functional groups (e.g., carboxyl, phenolic, amine groups).^161–166^ Although variable across environments,^162,164,167–177^ organic alkalinity has been detected even in low-DOM environments—including the open ocean, deep waters, and even certified reference materials—typically within 5 μmol kg^–1^.^154–156,177^ Methods such as NaOH back-titration have been developed to better quantify this contribution.^162,171,172,175,177^ While not yet routine, such methods are essential for improving carbonate system modeling and internal consistency.^9,123,141,152,154,155,165,166^

These internal consistency issues propagate directly into derived parameters, including calcium carbonate saturation states, sensitivity factors, and estimates of anthropogenic carbon. Errors in measured inputs, equilibrium constants, or inconsistent alkalinity definitions can amplify into substantial uncertainties in these higher-level diagnostics, which are central to quantifying ocean acidification and the ocean carbon sink. Resolving internal consistency is therefore not only a methodological requirement but also a prerequisite for robust estimation of natural, marine CO_2_ uptake and further mCDR approaches.

## 3 Oceanic Uptake of Anthropogenic CO_2_

Rising atmospheric CO_2_ from anthropogenic emissions has increased the air–sea CO_2_ concentration gradient, driving oceanic uptake as the carbonate system seeks to re-establish equilibrium.^8,178,179^ This persistent disequilibrium has increased surface-ocean CO_2_ concentrations (green in Figure 4) at rates closely tracking those observed in the atmosphere (purple in Figure 4).^180–190^ In this section, we assess the key processes controlling oceanic uptake, methods for quantifying anthropogenic CO_2_ storage, and the chemical feedbacks that emerge from this uptake.

**Figure 4:**
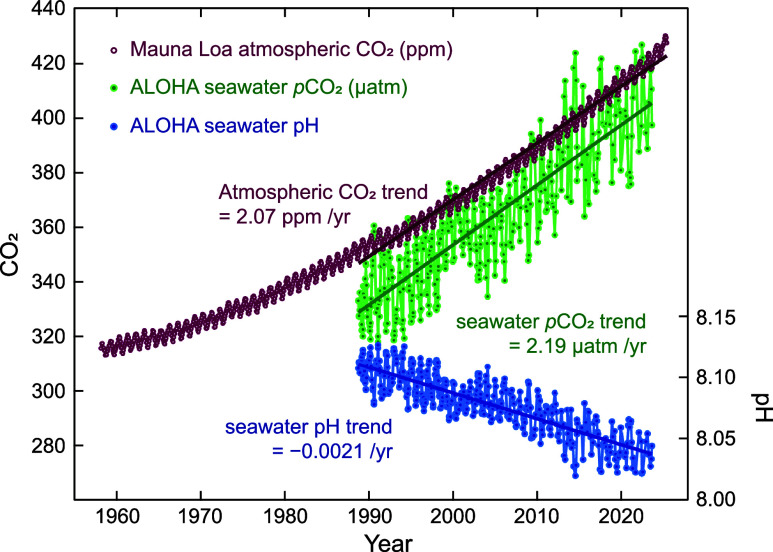
Temporal trends of atmospheric CO_2_, surface *p*CO_2_, and pH at Ocean Station ALOHA in the subtropical North Pacific. Adapted from ref 188. Copyright 2009 The Oceanography Society under Creative Commons CC BY license https://creativecommons.org/licenses/by/4.0/. Data updated with the most recent observations reported by ref 191.

### 3.1 Factors Influencing Oceanic CO_2_ Uptake and Distribution

Atmospheric CO_2_ is absorbed by the ocean across the air–sea interface, driven by the *f*CO_2_ gradient between surface seawater (sw) and the atmosphere (atm). The flux (F_CO2_) is expressed as

$$F_{\mathrm{CO}2}=K_{0}k_{\mathrm{CO}2}\left(f{\mathrm{CO}}_{2\left(\mathrm{sw}\right)}-f{\mathrm{CO}}_{2\left(\mathrm{atm}\right)}\right)$$ 14

where *K*_0_ is the solubility of CO_2_ in seawater, and *k*_CO2_ is the gas transfer velocity, primarily controlled by wind speed.^192,193^ While atmospheric CO_2_ rises steadily due to anthropogenic emissions, *f*CO_2(sw)_ in surface seawater shows large variability. This variability is locally governed by physical conditions, biogeochemical processes, and ocean circulation, making them the dominant controls on air–sea CO_2_ flux.

#### 3.1.1 Physical Drivers: The Solubility Pump

Temperature (*T*) and salinity (*S*) directly influence CO_2_ solubility (*K*_0_) and the dissociation constants (*K*_1_, *K*_2_) of carbonic acid, thereby modifying surface-ocean *f*CO_2_. A 1-unit increase in *S* raises *f*CO_2_ by ∼ 2.5%, whereas a 1 °C rise in *T* elevates *f*CO_2_ by ∼ 4%.^54^ Variations in *T* and *S* also alter seawater density, dictating circulation patterns (e.g., upwelling, deep-water formation) and modulating the transport of carbon-rich waters.

In most oceanic regimes, variability in *T* (up to 30 °C) exert a stronger control on *f*CO_2_ than variations in *S* driven by evaporation and precipitation (∼7 units). The combined influence of solubility and circulation constitutes the solubility pump, a process that stores anthropogenic CO_2_ on decadal to centennial time scales.^194,195^ Cold, high-latitude waters exemplify this mechanism: solubility enhancement reduces surface *f*CO_2_, favoring uptake from the atmosphere, while density increase drives deep-water formation, transporting absorbed CO_2_ to the ocean interior. Continuous replacement of surface waters by surrounding waters sustain this uptake capacity over time.

#### 3.1.2 Biological Drivers: The Soft-Tissue and Carbonate Pumps

Biological processes in the euphotic zone strongly influence surface *f*CO_2_ by altering carbon speciation:

$$106{\mathrm{CO}}_{2}+16{\mathrm{HNO}}_{3}+1H_{3}{\mathrm{PO}}_{4}+122H_{2}O↔{\left({\mathrm{CH}}_{2}O\right)}_{106}{\left({\mathrm{NH}}_{3}\right)}_{16}{\left(H_{3}{\mathrm{PO}}_{4}\right)}_{1}+138O_{2}$$ 15

$${\mathrm{Ca}}^{2+}+2{{\mathrm{HCO}}_{3}}^{-}↔{\mathrm{CaCO}}_{3}+H_{2}O+{\mathrm{CO}}_{2}$$ 16

Photosynthetic production of organic matter utilizes CO_2_, lowering surface *f*CO_2_ and enhancing atmospheric CO_2_ uptake (eq 15), whereas calcium carbonate (CaCO_3_) production releases CO_2_, raising *f*CO_2_ and opposing uptake (eq 16). The associated downward export and subsequent processing of these materials drive the soft-tissue pump and carbonate pump, respectively—biogeochemical mechanisms that regulate variability in surface *f*CO_2_.^194,195^

The long-term effect of these pumps depends on the fate of exported particles. Most organic matter and CaCO_3_ produced in the euphotic layer are respired or dissolved at depth, increasing DIC and TA, which can eventually re-enter the surface through overturning circulation.^195^ Only a small fraction of organic matter escapes remineralization at the seafloor, representing permanent storage on geological time scales.

#### 3.1.3 Chemical Buffering and Revelle Factor

Beyond physical and biological drivers, the chemical buffering capacity of seawater exerts a fundamental control on surface *f*CO_2_, and thus on air–sea CO_2_ exchange. This buffering is commonly expressed by the Revelle factor,^8,22,189,196,197^ which quantifies the sensitivity of surface *f*CO_2_ to changes in DIC:

$$\mathrm{Revelle factor}=\left(\Delta f{\mathrm{CO}}_{2}/f{\mathrm{CO}}_{2}\right)/\left(\Delta \mathrm{DIC}/\mathrm{DIC}\right)$$ 17

As CO_2_ accumulates in seawater, the Revelle factor increases, meaning that even small additions of DIC lead to disproportionately large rise in *f*CO_2_. This reduces the air–sea CO_2_ gradient and constrains further uptake. In contrast, regions with lower Revelle factor show weaker sensitivity and thus greater capacity for continued absorption. The Revelle factor is primarily governed by the ratio of DIC to TA and varies with *S*, *T*, and circulation patterns,^8,22,196^ adding a distinct chemical dimension to the physical and biological processes regulating oceanic CO_2_ uptake.

### 3.2 Quantification of Anthropogenic CO_2_ in the Ocean

Once absorbed by seawater, anthropogenic CO_2_ (C^ANT^) reacts to form carbonic acid, which rapidly dissociates into bicarbonate ions and protons, or combines with carbonate ions under higher pH conditions. This buffering reaction (eq 5) shifts the equilibrium toward bicarbonate ions. However, surface waters rarely equilibrate fully with the atmosphere before being subducted or advected into the ocean interior. During transport, their chemical properties such as DIC, TA, dissolved oxygen (O_2_), are modified by mixing with waters of different origin as well as biogeochemical transformations, including the remineralization of organic matter that produces regenerated carbon. This regenerated component adds to interior DIC and masks the anthropogenic signal, meaning that C^ANT^ in the ocean cannot be measured directly but must be inferred. Multiple approaches have been developed, including tracer-based frameworks such as transit time distribution and Green functions,^198–201^ isotopic constraints from the decline in oceanic ^13^C/^12^C ratio,^202–204^ large-scale ocean biogeochemical or data-assimilative circulation models.^205,206^ Among these, back-calculation methods and extended multiple linear regression have emerged as the most widely applied techniques and will be the focus of the following discussion.

#### 3.2.1 Back-Calculation Method

The back-calculation approach estimates C^ANT^ in a water parcel by separating it from the measured DIC (DIC^MEAS^) based on the parcel’s preformed conditions. This problem was first addressed by Brewer^207^ and Chen and Millero,^208^ with subsequent refinements providing more objective and reproducible frameworks.^209–213^ Conceptually, DIC^MEAS^ can be partitioned into:

$${\mathrm{DIC}}^{\mathrm{MEAS}}={\mathrm{DIC}}^{\mathrm{PRE}}+{\mathrm{\Delta C}}^{\mathrm{BIO}}$$ 18

where DIC^PRE^ is the preformed DIC concentration at the time of last atmospheric contact, while ΔC^BIO^ accounts for remineralization of organic matter and dissolution of CaCO_3_. DIC^PRE^ can further be decomposed into three terms:

$${\mathrm{DIC}}^{\mathrm{PRE}}={{\mathrm{DIC}}^{\mathrm{PRE}}}_{\mathrm{PI}}+C^{\mathrm{ANT}}+{\mathrm{\Delta C}}^{\mathrm{DISEQ}}$$ 19

where DIC^PRE^_PI_ is the preindustrial equilibrium concentration, C^ANT^ is anthropogenic carbon addition, and ΔC^DISEQ^ is a diagnostic residual reflecting incomplete air–sea equilibration at the time of water mass formation. Accordingly:

$$C^{\mathrm{ANT}}={\mathrm{DIC}}^{\mathrm{MEAS}}-{\mathrm{\Delta C}}^{\mathrm{BIO}}-{{\mathrm{DIC}}^{\mathrm{PRE}}}_{\mathrm{PI}}-{\mathrm{\Delta C}}^{\mathrm{DISEQ}}$$ 20

The biological correction ΔC^BIO^ is derived from deviations in O_2_ and TA relative to preformed values:

$${\mathrm{\Delta C}}^{\mathrm{BIO}}=R_{C:O2}\left({O_{2}}^{\mathrm{MEAS}}-{O_{2}}^{\mathrm{PRE}}\right)+0.5\left[\left({\mathrm{TA}}^{\mathrm{MEAS}}-{\mathrm{TA}}^{\mathrm{PRE}}\right)+R_{N:O2}\left({O_{2}}^{\mathrm{MEAS}}-{O_{2}}^{\mathrm{PRE}}\right)\right]$$ 21

where O_2_^PRE^ is assumed to be saturated at the surface.^214–216^ *R*_C:O2_ and *R*_N:O2_ are stoichiometric ratios linking carbon (C) and nitrogen (N) to O_2_ (Redfield ratio^217^ in eq 15).^218–221^ TA^PRE^ is estimated using multiple linear regression with conservative tracers (e.g., *S* and oxygen-nutrient combinations).^209–213,222–225^ DIC^PRE^_PI_ is then calculated from equilibrium concentration at given *S*, *T*, and TA^PRE^, assuming a preindustrial CO_2_ of 280 μatm (see Section 2.4.1).

To constrain ΔC^DISEQ^, Gruber et al.^209^ introduced a quasi-conservative tracer C*:

$$C^{*}=\mathrm{DIC}-C^{\mathrm{BIO}}$$ 22

which is invariant to transport and biological cycling, leaving air–sea exchange as the dominant control.^195,209,226^ Deviations from preindustrial baseline define:

$${\mathrm{\Delta C}}^{*}={\mathrm{DIC}}^{\mathrm{PRE}}-{{\mathrm{DIC}}^{\mathrm{PRE}}}_{\mathrm{PI}}=C^{\mathrm{ANT}}+{\mathrm{\Delta C}}^{\mathrm{DISEQ}}$$ 23

In deep, old waters where C^ANT^ is negligible, ΔC* approximates ΔC^DISEQ^. For more recently ventilated waters, ΔC^DISEQ^ can be estimated from tracer-derived water ages (e.g., CFCs, SF_6_, radioactive tracers):

$${\mathrm{\Delta C}}^{\mathrm{DISEQ}}={{\mathrm{\Delta C}}_{\mathrm{TIME}}}^{*}={\mathrm{DIC}}^{\mathrm{PRE}}-{{\mathrm{DIC}}^{\mathrm{PRE}}}_{\mathrm{TIME}}$$ 24

where DIC^PRE^_TIME_ is the equilibrium concentration at the time of last ventilation. For water masses formed by mixing, fraction-weighted averages of ΔC* and ΔC_TIME_* yield robust C^ANT^ estimates.^209–213^

Back-calculation methods based on ΔC* generally yield global C^ANT^ storage estimates with uncertainties of 7–20%.^8,205,213^ Subsequent refinements have addressed biases associated with incomplete O_2_ saturation, fixed stoichiometric ratios, linear mixing assumptions, and uncertainties in reconstructing preformed properties.^216,227–232^ Nonetheless, the method remains fundamentally limited by the assumption that the natural carbon cycle has been in steady state since preindustrial times, a simplification that introduces uncertainties in the reconstruction of preindustrial baselines, especially ΔC^DISEQ^.^233^

#### 3.2.2 Extended Multiple Linear Regression Method

An alternative to back-calculation is the extended multiple linear regression (eMLR) method, which estimates temporal changes in C^ANT^ (ΔC^ANT^) from repeat hydrographic surveys.^234–241^ Unlike back-calculation, eMLR does not require explicit assumptions about preindustrial properties. Instead, it partitions observed changes in DIC between two occupations, *t*_1_ and *t*_2_, in anthropogenic and natural components:

$$\mathrm{\Delta DIC}\left(t_{2}-t_{1}\right)={\mathrm{\Delta C}}^{\mathrm{ANT}}\left(t_{2}-t_{1}\right)+{\mathrm{\Delta C}}^{\mathrm{NAT}}\left(t_{2}-t_{1}\right)$$ 25

where ΔC^NAT^ reflects natural variability from circulation, mixing, biological processes, and air–sea gas exchange. Using the quasi-conservative tracer C*, which corrects for biological signals^195,209,226^ as Equation 22, the change can be written as

$${\mathrm{\Delta C}}^{*}\left(t_{2}-t_{1}\right)={\mathrm{\Delta C}}^{\mathrm{ANT}}\left(t_{2}-t_{1}\right)+{{\mathrm{\Delta C}}^{\mathrm{NAT}}}_{\mathrm{gasex}}\left(t_{2}-t_{1}\right)$$ 26

with ΔC^NAT^_gasex_ representing the residual natural signal from air–sea CO_2_ exchange.^195,235^

Because repeat surveys span years to decades, observations are first normalized to a reference year *t*_ref_ using the transient steady state concept.^241,242^ This concept assumes, under the exponential rise of atmospheric CO_2_ compared to ocean’s equilibration, the vertical C^ANT^ profiles remain nearly constant, scaling proportionally with atmospheric growth. Thus, any C* observation at time *t* is adjusted^235^ as

$$C^{*}\left(t_{\mathrm{ref}}\right)=C^{*}\left(t\right)-\beta \left(t\right)\cdot C^{\mathrm{ANT}}\left(t_{\mathrm{ref}}\right)$$ 27

where C^ANT^(*t*_ref_) is obtained from independent estimates (e.g., back-calculation in Section 3.2.1) and β­(*t*) represents the relative atmospheric CO_2_ change between the *t* and *t*_ref_:

$$\beta \left(t\right)=\left(f{\mathrm{CO}}_{2\left(\mathrm{atm}\right)}\left(t\right)-f{\mathrm{CO}}_{2\left(\mathrm{atm}\right)}\left(t_{\mathrm{ref}}\right)\right)/f{\mathrm{CO}}_{2\left(\mathrm{atm}\right)}\left(t_{\mathrm{ref}}\right)$$ 28

Once normalized, the adjusted C* data are regressed against a suite of independent tracers (*T*, *S*, O_2_, nutrients):

$$C^{*}\left(t_{\mathrm{ref}}\right)=a_{0}+\sum a_{i}\cdot T_{i}\left(t\right)+residual\left(t\right)$$ 29

where *a*_0_ and *a*_*i*_ are regression coefficients. The extended method^235,240^ then quantifies ΔC^ANT^ by evaluating differences in regression coefficients between survey periods, which effectively minimizes ΔC^NAT^_gasex_ in Equation 26:

$${\mathrm{\Delta C}}^{\mathrm{ANT}}\left(t_{2,\mathrm{ref}}-t_{1,\mathrm{ref}}\right)=\left(a_{2,0}-a_{1,0}\right)+\sum \left(a_{2,i}-a_{1,i}\right)\cdot {T^{\mathrm{clim}}}_{i}$$ 30

with *T* ^clim^_*i*_ representing the climatological distributions of the tracers.^235^

The accuracy of the eMLR­(C*) method depends on predictor choice and data spatial coverage.^238,243–245^ At global and basin scales, uncertainties are typically ± 10%, but increase to ± 20% at sub-basin scales due to greater variability in circulation and biogeochemical processes.^235–237,245–247^ Moreover, the method assumes a transient steady state for C^ANT^, a simplification that may not hold in regions with rapid dynamical changes. Despite these caveats, the eMLR­(C*) method remains one of the most comprehensive methods for quantifying changes in anthropogenic carbon storage across all major ocean basins depth ranges.

### 3.3 Anthropogenic CO_2_ in the Ocean

The distribution of C^ANT^ is primarily governed by transport along isopycnals, which determine how effectively surface signals are carried down into the ocean interior.^205,206,248^ Mode waters formed at mid- to high-latitudes ventilate on decadal time scales, rapidly transferring C^ANT^ into the upper thermocline. In temperate convergence zones, thick sloping isopycnals near potential density of ∼ 26 kg m^–3^ act as major pathways for recently ventilated, C^ANT^-enriched waters.^8,237^ By contrast, denser surfaces (e.g., ∼ 27 kg m^–3^) exchange more slowly, and their C^ANT^ signals remain weak, often detectable only near surface outcrops.^8,237^

C^ANT^ penetration depths and inventories vary markedly among basins (Figure 5), reflecting differences in circulation and ventilation. The North Atlantic, where active deep-water formation drives ΔC^ANT^ exceeding 5 μmol kg^–1^ below 1,000 m between 1994 and 2007 (Figure 5), accounts for ∼ 23% of the global inventory despite occupying only ∼ 15% of the ocean area (see Figure 6a and b).^8,237^ The Southern Ocean is another major entry site, but south of 50°S strong upwelling of carbon-rich preindustrial waters dilutes the anthropogenic signal, limiting its share to ∼ 9% of the global total.^8,200,206,237^ In the North Pacific, which lacks deep-water formation, C^ANT^ penetration is confined largely to intermediate depths through subducted waters (Figure 5), giving the basin only about half the Atlantic areal storage (Figure 6a and b).^8,237^

**Figure 5:**
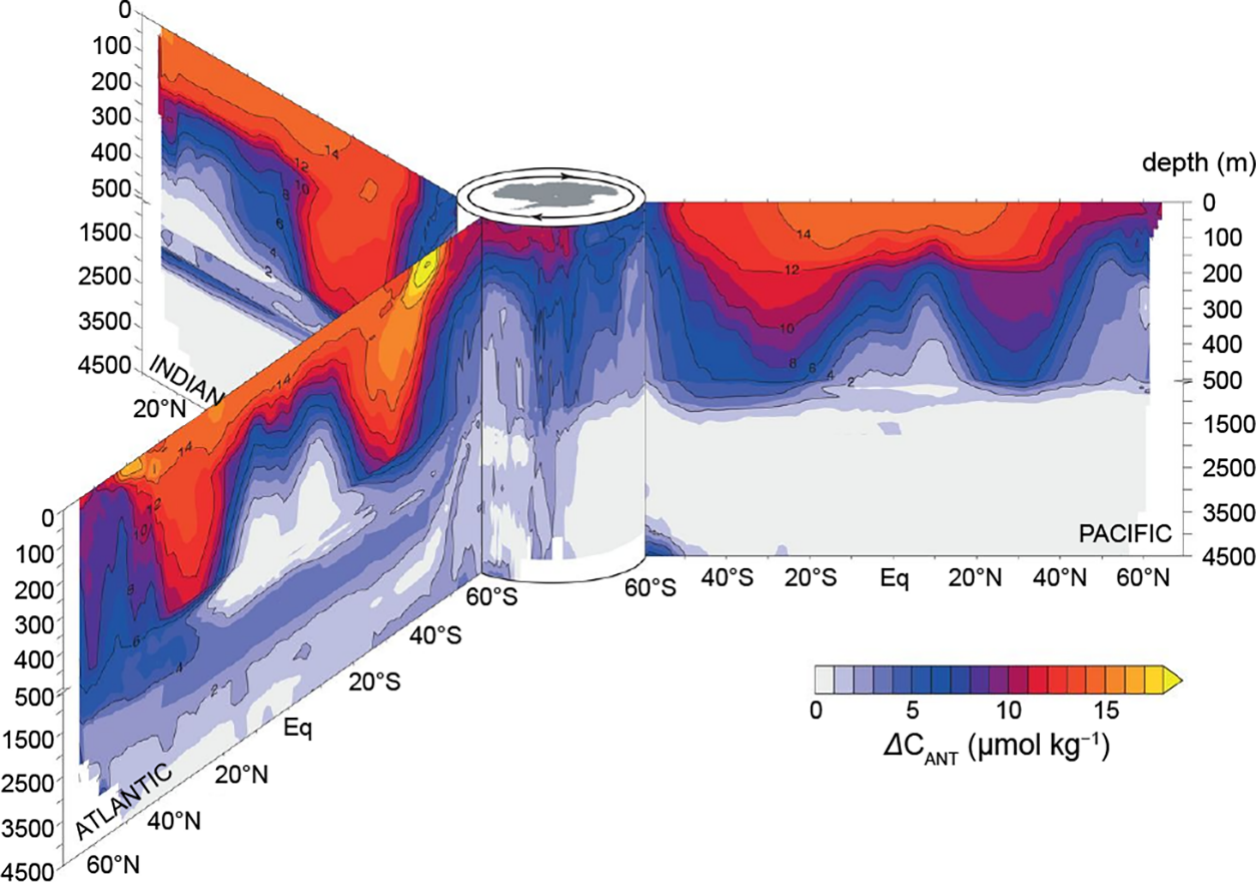
Meridional sections showing changes in C^ANT^ (ΔC^ANT^) in the Atlantic, Pacific, and Indian Oceans between 1994 and 2007. Reproduced with permission from ref 237. Copyright 2019 The American Association for the Advancement of Science.

**Figure 6:**
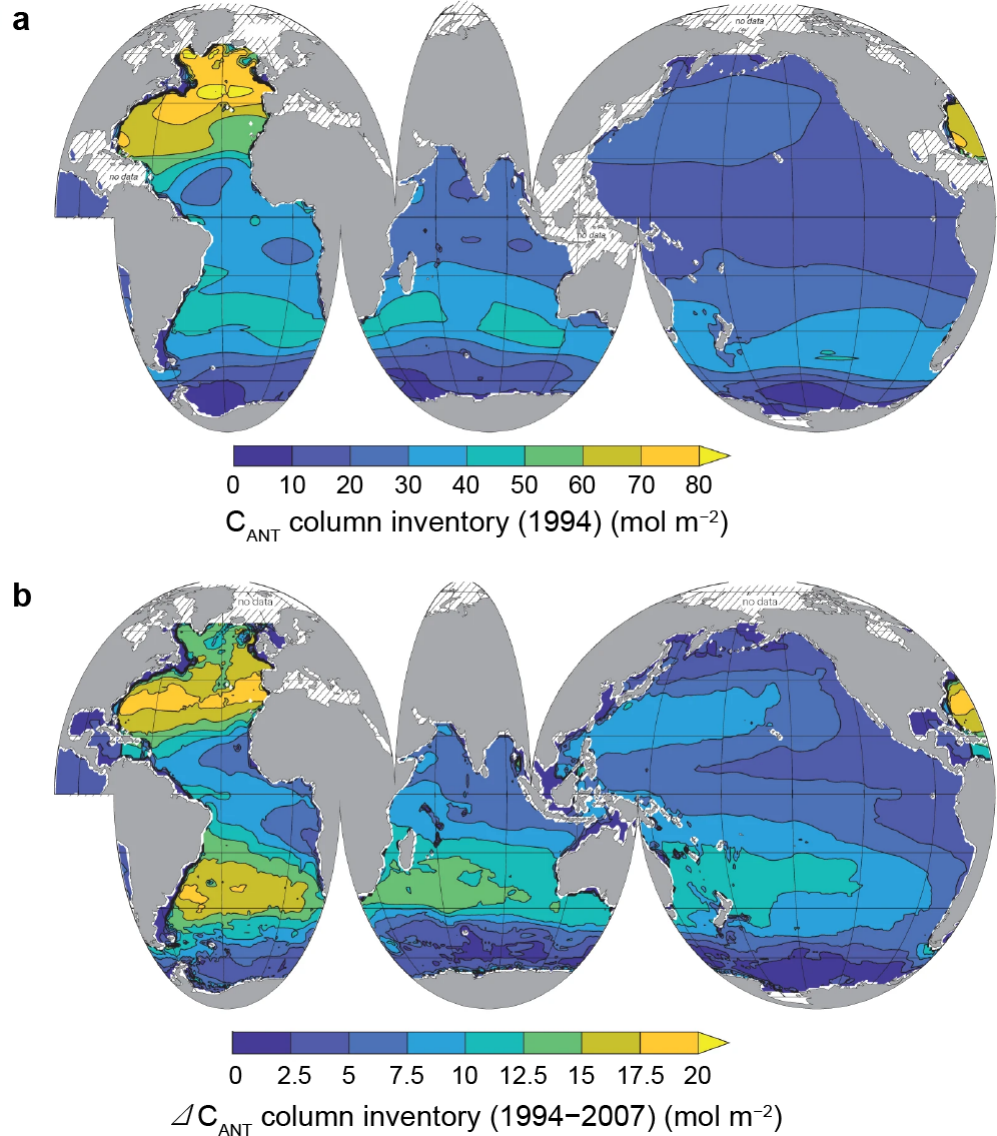
(**a**) Column inventory of C^ANT^ for 1994 and (**b**) water-column ΔC^ANT^ between 1994 and 2007 for the global ocean. Reproduced with permission from ref 249. Copyright 2023 Springer Nature.

Globally, C^ANT^ is concentrated in the upper ocean because equilibration with the atmosphere occurs rapidly at the surface (Figure 5), whereas transfer to the interior rather follows decadal-to-centennial circulation pathways. By the mid-1990s, about 50% of global C^ANT^ resided above 400 m, with detection depths averaging 1,000 m.^8^ By 2007, the signal had expanded vertically: ∼ 75% of the ΔC^ANT^ remained above 1,000 m, and only ∼ 7% reached below 2,000 m.^237^ Increases were especially strong in the upper 100 m, where ΔC^ANT^ rose by 14 μmol kg^–1^, while gains at depth were comparatively modest.^237^

Taken all C^ANT^ inventories, the global ocean has absorbed about 30% of annual anthropogenic emissions, although the uptake rate varied over time.^2,200,205,237,250–254^ Oceanic storage averaged 2 Pg C year^–1^ during the 1990s and increased to ∼ 3 Pg C year^–1^ in recent decades, consistent with accelerating atmospheric CO_2_ growth.^249^ Cumulative inventories have risen from 118 ± 19 Pg C by the mid-1990s to 152–161 Pg C by 2010 and are currently estimated at 185 ± 35 Pg C as of 2024.^2,8,205,206,237^ These figures highlight the central role of the ocean as the largest long-term sink moderating atmospheric CO_2_ accumulation.^2^

### 3.4 Feedbacks on Carbon Uptake and Future Ocean Carbon Sink

Although rising atmospheric CO_2_ continues to drive air–sea exchange, the ocean’s capacity to absorb CO_2_ is projected to weaken in decades to come.^233,249,255^ This decline reflects how combined climate-induced changes in physics, chemistry, biology diminish the efficiency of marine carbon uptake.

From a physical standpoint, surface warming reduces CO_2_ solubility, raising surface-ocean *p*CO_2_ and thereby weakening the air–sea gradient; in some regions, this shift may even reverse fluxes from net uptake to net outgassing.^233,256^ Warming and freshening from ice melt also intensify stratification and reduce ventilation, slowing the transfer of carbon into the ocean interior.^15^

Chemical feedbacks further constrain uptake. Increasing atmospheric CO_2_ lowers surface-ocean pH and elevates the Revelle factor, amplifying sensitivity to additional CO_2_ inputs.^188,190^ This trend, projected to increase the Revelle factor by 34% by 2100,^13,15^ is exacerbated by stratification, which limits the upward supply of alkalinity and weakens ocean buffering capacity.^15^ Declining carbonate ion concentrations reduce biogenic calcification and associated CO_2_ release at the surface, while shoaling the saturation horizon, enhancing CaCO_3_ dissolution at depth, and altering alkalinity cycling. The net effect of these processes for air–sea CO_2_ exchange remains uncertain.^257–259^

Biological feedbacks add further complexity. Elevated CO_2_ may stimulate photosynthesis in some phytoplankton groups,^233,260^ yet intensified stratification generally suppresses nutrient supply to the euphotic zone, weakening the biological pump.^14,261^ Large-scale nutrient redistribution under continued warming is expected to lower upper-ocean primary production and reduce organic carbon export by up to 40%, further diminishing the biological contribution to carbon storage.^261^

Taken together, these interacting feedbacks indicate that the future ocean carbon sink will remain highly sensitive to climate forcing, potentially reinforcing positive carbon–climate feedbacks. This emphasizes both the urgency of rapid emission reductions and complementary need for mCDR strategies to help sustain long-term climate stabilization.

## 4 Ocean Alkalinity Enhancement as a Pathway for CO_2_ Removal and Storage

### 4.1 Overview and Introduction to Ocean Alkalinity Enhancement

Ocean alkalinity enhancement (OAE) introduces alkaline substances to seawater so that the ocean’s alkalinity reservoir buffers more CO_2_ and enhances atmospheric CO_2_ uptake.^9,262^ Because seawater contains ∼ 2.4 mM TA and ∼ 2 mM DIC, even modest chemical shifts significantly increase carbon storage. For example, a 0.1 unit increase in surface ocean pH across global exclusive economic zones could remove over 1 Pg of CO_2_ annually,^263^ potentially representing a lower bound of OAE capacity.^264,265^

The chemistry behind OAE is covered extensively elsewhere,^262,266^ and thus is reviewed briefly here. The OAE process consists of two stages (Figure 7): (1) alkalinity transfer from added material to seawater (green arrow), and (2) subsequent CO_2_ absorption from the atmosphere (blue arrow). Added bases such as OH^–^ shift the carbonate equilibrium toward bicarbonate and carbonate ions:

$${\mathrm{CO}}_{2}^{*}+{\mathrm{OH}}^{-}↔{{\mathrm{HCO}}_{3}}^{-}$$ 31

$${{\mathrm{HCO}}_{3}}^{-}+{\mathrm{OH}}^{-}↔{{\mathrm{CO}}_{3}}^{2-}+H_{2}O$$ 32

**Figure 7:**
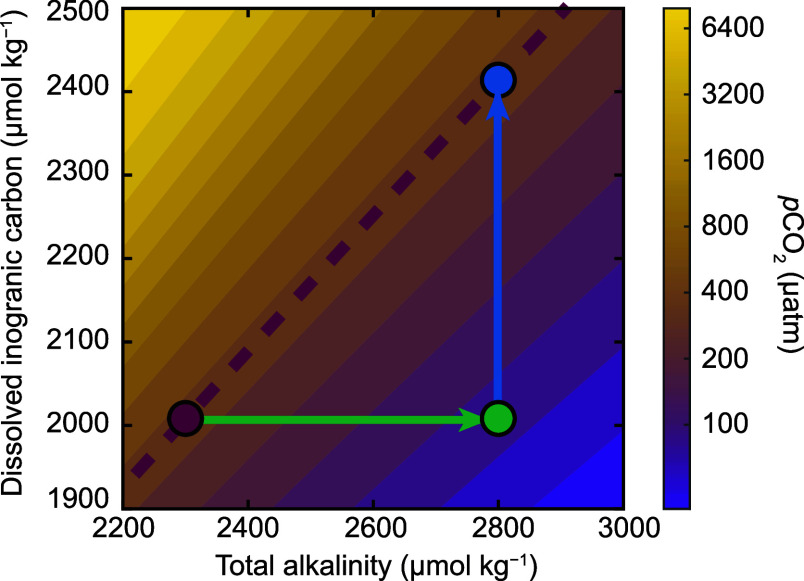
A contour plot of seawater *p*CO_2_ as a function of TA and DIC. The atmospheric value of 420 μatm is displayed as a dashed line. Starting with a typical seawater composition (purple dot), OAE introduces alkalinity to seawater along the green arrow, resulting in seawater with decreased *p*CO_2_ (green dot). This seawater parcel then takes up CO_2_ from the atmosphere (blue arrow) until it reaches equilibrium, resulting in increased DIC (blue dot). These combined arrows represent the CO_2_ removal (CDR) process associated with OAE.

These reactions do not change total DIC but lower surface *p*CO_2_, enhancing the air–sea CO_2_ gradient and driving greater CO_2_ uptake (see eq 14), which ultimately increases the oceanic DIC inventory. OAE stores carbon as bicarbonate and carbonate ions, which do not provoke additional acidification typically associated with oceanic CO_2_ uptake.^190^ In some cases, OAE could provide a cobenefit of locally mitigating ocean acidification.^267,268^

The foundational understanding of OAE stems from decades of research on weathering and global carbon cycling,^269–271^ now reframed in the context of accelerated weathering for climate mitigation. Building on this foundation, this section reviews the core chemistry of OAE—including alkalinity generation, transfer to seawater, and CO_2_ removal (CDR)—focusing on three key aspects: (1) current methods for producing and delivering alkalinity to seawater (Section 4.2); (2) open-system CO_2_ exchange processes (Section 4.3); and (3) monitoring, reporting, and verification, as well as environmental safety (Section 4.4).

### 4.2 Alkalinity Production and Transfer to Seawater

Several methods are currently being explored for delivering alkaline materials to seawater, including the mining, grinding, and dispersal of minerals into the marine environment (Figure 8).^266^ This section focuses on five common materials for OAE: olivine (Mg_(2–*x*)_Fe_*x*_SiO_4_), calcium carbonate (CaCO_3_) (e.g., calcite, aragonite), brucite or magnesium hydroxide (Mg­(OH)_2_), calcium oxide and hydroxide (CaO, Ca­(OH)_2_), and sodium hydroxide (NaOH) (Table 2). All these minerals generate alkalinity when dissolved in seawater, though they differ in composition, solubility, reaction kinetics, and side reactions. Potential feedbacks to OAE include secondary mineral precipitation, which can occur on particle surfaces,^272^ within the water column under altered chemical conditions,^273,274^ or at larger scales as ocean responses to enhanced TA.^275^

**Figure 8:**
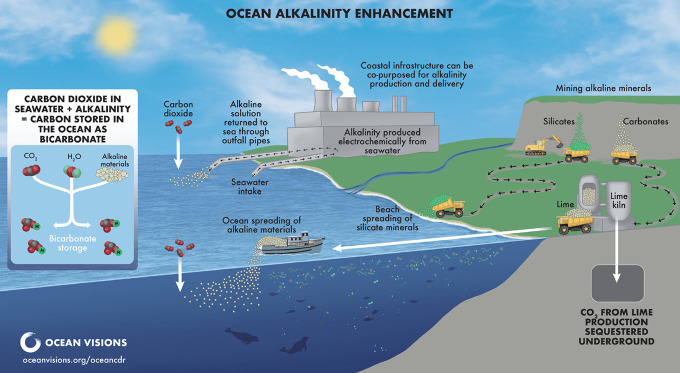
Illustration of potential approaches for OAE. Reproduced from Ocean Visions, Copyright 2023 Ocean Visions under Creative Commons CC BY-NC-ND license https://creativecommons.org/licenses/by-nc-nd/4.0/.

**Table 2:** Current Commonly Considered Materials for OAE

Material	Effective alkalinity yield (mol alkalinity g^–1^)^a^	maximum soluble pH^g^	log_10_R_diss_ (mol m^–2^ s^–1^)	Secondary reactions
Olivine (Mg_(2–x)_Fe_*x*_SiO_4_)^b^	0.020–0.028	14^h^	10^–7.5^–10^–10.7^	Serpentinite, clays, iron minerals, (Ca,Mg)CO_3_
Calcium Carbonate (CaCO_3_)^c^	0.012^d^	7.4	10^–9.5^–10^–4^	(Ca,Mg)CO_3_
Brucite (Mg(OH)_2_)^e^	0.035	10.4	10^–7^–10^–2.5^	(Ca,Mg)CO_3_
Slaked Lime (CaO) and/or Ca(OH)_2_)^f^	0.025–0.03	12.3	10^–4^–10^–3^	(Ca,Mg)CO_3,_ Mg(OH)_2_
Sodium Hydroxide (NaOH)	0.025	>14^h^	instantaneous	(Ca,Mg)CO_3,_ (Ca,Mg)(OH)_2_

a Effective alkalinity yield was calculated using Equations 33–38 and molar mass for each material.

b Olivine data were extracted from refs 277 and 278.

c CaCO_3_ solubility was taken from ref 279 and dissolution rate (R_diss_) from refs 280 and 281.

d For CaCO_3_, a neutralization factor of 0.56 ^276^ is applied due to CO_3_^2–^ production rather than OH^–^ (eq 34).

e Mg­(OH)_2_ data were taken from refs 282 and 283.

f Ca­(OH)_2_ solubility was taken from ref 284 and R_diss_ from ref 285.

g Solubility limits were calculated at *T* = 18 °C, *S* = 32, [Mg^2+^] = 53 mmol kg^–1^, and [Ca^2+^] = 10.2 mmol kg^–1^, by determining the [OH^–^] concentration at which each mineral becomes saturated.

h Olivine and NaOH were assumed to be soluble over pH 0–14.

#### 4.2.1 Effective Alkalinity Yield

Alkalinity is introduced to seawater through the dissolution of alkaline materials (Table 2), which consumes protons and increases TA while maintaining charge balance:

$${\mathrm{Mg}}_{\left(2-x\right)}{\mathrm{Fe}}_{x}{\mathrm{SiO}}_{4}\to \left(2-X\right){\mathrm{Mg}}^{2+}+X{\mathrm{Fe}}^{2+}+H_{4}{\mathrm{SiO}}_{4}+4{\mathrm{OH}}^{-}$$ 33

$${\mathrm{CaCO}}_{3}\to {\mathrm{Ca}}^{2+}+{{\mathrm{CO}}_{3}}^{2-}$$ 34

$$\mathrm{Mg}{\left(\mathrm{OH}\right)}_{2}\to {\mathrm{Mg}}^{2+}+2{\mathrm{OH}}^{-}$$ 35

$$\mathrm{Ca}{\left(\mathrm{OH}\right)}_{2}\to {\mathrm{Ca}}^{2+}+2{\mathrm{OH}}^{-}$$ 36

$$\mathrm{CaO}+H_{2}O\to {\mathrm{Ca}}^{2+}+2{\mathrm{OH}}^{-}$$ 37

$$\mathrm{NaOH}\to {\mathrm{Na}}^{+}+{\mathrm{OH}}^{-}$$ 38

Stoichiometric alkalinity production per gram of mineral, assuming complete dissolution, ranges from 0.012 mol alkalinity g^–1^ for CaCO_3_ to 0.035 mol alkalinity g^–1^ for Mg­(OH)_2_ (Table 2). Olivine releases 4 mol of OH^–^ per mole but has a moderate yield by weight due to its high molar mass. CaCO_3_ dissolution is distinct in producing carbonate alkalinity (CO_3_^2–^), which also increases the DIC pool. However, because CO_3_^2–^ is a weaker base than OH^–^, its buffering effect—and thus effective alkalinity yield—is about 56% lower under surface ocean conditions.^276^

In addition to alkalinity, the dissolution of these materials releases cations and other constituents. For example, olivine releases iron and silicic acid (eq 33) and may also release trace metals such as nickel, depending on its composition.^286^ These trace elements can influence dissolved metal and silica budgets and may trigger biological responses—beneficial or harmful.^287^ The oxidation of reduced iron released during olivine dissolution would also produce acidity, thereby lowering the net alkalinity yield.^286^

Naturally occurring Mg­(OH)_2_ is rare at the Earth’s surface;^288^ however it offers the highest alkalinity yield due to its low molar mass. Industrial products CaO and Ca­(OH)_2_ are derived from the calcination of limestone (i.e., CaCO_3_),^289^ a process that emits CO_2_. To ensure carbon negativity, CO_2_ generated via calcination must be captured and stored (Figure 8). NaOH is typically produced via the chlor-alkali process, which generates equimolar HCl or Cl_2_ as byproducts. Emerging methods propose in situ electrolysis of seawater (NaCl solution) to generate NaOH while extracting HCl.^266^ These production pathways and their life-cycle emissions are further discussed in Section 4.4.

#### 4.2.2 Solubility

NaOH, unlike solid minerals, is unstable as a solid, hygroscopic, and highly soluble in water—up to 50 wt % (∼19 mol L^–1^). Other solid alkaline materials exhibit a range of solubilities, typically described by their saturation state (Ω), defined as the ratio of the in situ ion activity product (IAP) to the mineral’s solubility product (*K*_sp_):

$$\Omega =\mathrm{IAP}/K_{\mathrm{sp}}$$ 39

Ω depends on ionic strength, *T*, and *P*, as well as pH, and the concentrations of dissolved magnesium, calcium, iron, and silica. Seawaters are undersaturated with respect to olivine, making it soluble across all pH values.^277^ In contrast, CaCO_3_ minerals are sparingly soluble in seawater due to their low solubility and the high background concentration of calcium (∼10.2 mM).^279^ Because CO_3_^2–^ concentrations are pH-dependent, CaCO_3_ becomes more soluble at pH ≤ 7.4—conditions uncommon in surface waters but typical in deep oceans, sediment porewaters, marine snow aggregates, or zooplankton guts.^290–293^

Both Mg­(OH)_2_ and Ca­(OH)_2_ have relatively high solubility in seawater, as indicated by the higher pH needed for saturation compared with olivine and CaCO_3_ (Table 2). However, their solubility product values and sensitivities to *T*, *P*, and ionic strength remain poorly constrained, partly due to coprecipitation with CaCO_3_ under DIC-rich conditions.^272^ Given the growing interest in these compounds for OAE applications,^268,294^ further laboratory studies are needed to refine their solubility parameters and thermodynamic behavior.

#### 4.2.3 Dissolution Kinetics

Assessing the effectiveness of OAE requires consideration of both the solubility of the added materials and their dissolution rates (R_diss_; mol m^–2^ s^–1^) (Table 2). In general, dissolution rate increases as seawater Ω decreases, which often corresponds to lower pH (Figure 9).^295,296^ Under highly undersaturated, low-pH conditions, interfacial transport and diffusion, rather than surface reaction kinetics, become the primary controls on the process.^297^ The relative importance of transport versus surface control depends on mixing, fluid exchange, and intrinsic reaction rates (Figure 9).^298,299^

**Figure 9:**
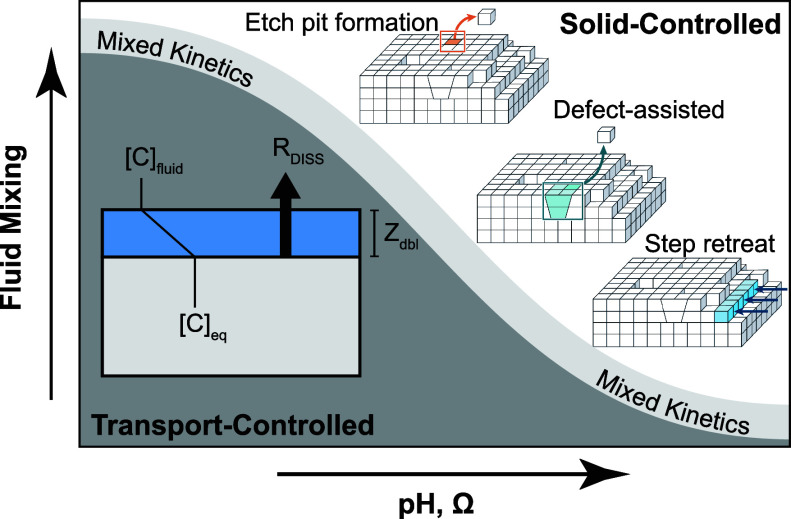
Schematic of the relationship between fluid mixing and transport control (dark gray area)­and surface-side mineral dissolution control (white area). For solid-controlled reactions, as saturation state increases toward equilibrium, typical reaction mechanisms progress from 2-D etch pit formation at deep undersaturation, to dissolution at surface defects, and finally, very near equilibrium, dissolution only at step edges.^296,300^ As undersaturation deepens, reaction rates increase, and fluid transport away from the mineral surface becomes increasingly important to characterize. The end-member transport scenario is within pore fluids, where the entire system is completely diffusively controlled.

Some materials, notably CaCO_3_, exhibit highly nonlinear dissolution kinetics with respect to Ω.^301^ This behavior has been explained using crystal growth theory,^302^ which links dissolution rates to surface energetics. Near equilibrium, only high-energy sites—such as screw dislocations and step edges—remain reactive.^296^ As Ω and pH decrease, larger areas of the mineral surface become active, progressing from the dissolution of defects to the formation and propagation of etch pits (Figure 9).^303^ This theory also explains why surface roughness correlates with higher dissolution rates: rougher surfaces expose more high-energy sites.^278^

Although CaCO_3_ minerals rarely encounter strong undersaturation in the ocean, bulk seawater is typically undersaturated with respect to olivine, Mg­(OH)_2_, and Ca­(OH)_2_ (Table 2). These materials are therefore expected to dissolve mainly through etch pit propagation, making surface speciation and transport processes critical rate-limiting factors.^282^ In situ dissolution may also be inhibited by adsorbed ions such as magnesium,^304^ sulfate,^300^ phosphate,^305,306^ and organic matter.^307^ While olivine dissolution as a function of pH is relatively well characterized,^278^ the literature is more limited for Mg­(OH)_2_ ^282,308^ and Ca­(OH)_2._ ^309,310^ To optimize OAE efficacy and better understand alkalinity transfer, further research is needed on the dissolution rate of these materials under oceanic conditions.

#### 4.2.4 Fluid and Sediment Transport Considerations

Far from equilibrium, when dissolution rates are high, solute transport becomes the major rate-limiting step (Figure 9). This is typically governed by diffusion across a diffusive boundary layer (DBL), which can become saturated with respect to the dissolving material.^297^ DBL thickness and transport rates can be experimentally assessed using rotating disk setups.^285,299^ The DBL regulates both the removal of dissolved products from, and proton delivery to, the mineral surface.^281^ Transport control is especially critical in sediment environments, where confined conditions—such as within porewaters or at the sediment-water interface—further constrain dissolution.^277,290^ Here, factors like diffusion, speciation, bioturbation, bioirrigation, and DBL dynamics all influence alkalinity fluxes.^311^

Sediment heterogeneity and reaction product buildup add complexity, requiring empirical validation through laboratory solubility and rate measurements, along with parametrization of key physical and biological processes. Incorporating biological processes such as aerobic and anaerobic respiration is essential for accurately reproducing sediment mineral behaviors and properties.^312–314^ Advances in open-access sediment-porewater models are improving predictions of alkalinity fluxes and should be further developed for OAE applications.^315^

For OAE-driven CDR to be effective, the generated alkalinity must reach the surface mixed layer, where it can lower surface-ocean *p*CO_2_ and enhance CO_2_ uptake from the atmosphere. In coastal environments, sediment transport may play a critical role, as wave and tidal processes can redistribute alkaline amendments away from deployment sites.^277,316^ As sediment-based OAE scales up, the development of robust tracking methods and sediment transport models will be essential to verify amendment dispersion and placement strategies, and to assess biogeochemical impacts.

Large-scale mineral amendments must be assessed within the framework of global sediment budgets. Removing 1 Pg of CO_2_ annually requires processing of 22.7 Tmol of CO_2_, which entails 27 Tmol of alkalinity addition, assuming a CDR efficiency of 0.85 mol per mol of alkalinity (see Section 4.3). Given olivine’s alkalinity yield (0.02–0.028 mol alkalinity g^–1^; Table 2), this translates to 0.96–1.35 Pg of olivine amendments annually—comparable to Europe’s and Africa’s total sediment delivery and ∼ 30% of North America’s.^317^ For materials like CaCO_3_ with lower alkalinity yields, required sediment additions would be even greater.

These sediment amendment estimates assume complete dissolution and full transfer of added alkalinity to the surface ocean—idealized scenarios unlikely to be achieved in practice. Therefore, they represent lower bounds on amendment requirements. At these scales, mineral additions could significantly alter coastal sediment composition and large-scale transport dynamics, especially when using slow-dissolving materials like olivine and CaCO_3_. These impacts must be carefully assessed as OAE implementation expands.

#### 4.2.5 Secondary Reactions and Alkalinity Consumption

The efficiency of alkalinity transfer into seawater depends on dissolution, transport, and feedbacks from mineral precipitation. Among these, CaCO_3_ precipitation is the most significant, releasing ∼ 0.6 mol of CO_2_ per mol formed by shifting the carbonate system toward CO_2_ through CO_3_^2–^ consumption.^318^ Although surface seawaters are generally supersaturated with respect to CaCO_3_, abiotic precipitation is kinetically limited and typically requires a nucleation threshold, either via seed minerals or homogeneous nucleation.^272,274,319–321^ Similar constraints likely apply to more soluble materials like Mg­(OH)_2_, CaO, and Ca­(OH)_2_, although their high solubility means they have not yet been thoroughly studied in this context. In the case of olivine, dissolution produces silicic acid and trace metals (e.g., Fe^2+^; eq 33), which may lead to secondary mineral formation and/or oxidation that consumes alkalinity.^277,322^ The extent of such feedbacks depends on local supersaturation, the presence of other materials, and the degree of fluid mixing with ambient seawater.

The risk of alkalinity loss via precipitation is highest for highly soluble materials such as NaOH, Ca­(OH)_2_, and Mg­(OH)_2_ (Table 2). These materials require vigorous mixing to avoid rapid precipitation.^263,323^ For example, NaOH addition may trigger the formation of Ca­(OH)_2_ or Mg­(OH)_2_ due to seawater’s high Ca^2+^ and Mg^2+^ concentrations. While Ca­(OH)_2_ dissolves rapidly and is unlikely to persist in the water column,^323^ Mg­(OH)_2_ may settle below the surface mixed layer before redissolving, resulting in alkalinity loss from the liquid phase and reduced CO_2_ uptake efficiency. Similar feedbacks due to Mg­(OH)_2_ formation and redissolution may occur during ocean liming, though their overall importance appears limited.^272,309^ CaCO_3_ precipitation is a universal risk across OAE materials, particularly because precipitated CaCO_3_ is not readily redissolved under surface ocean conditions. Experimental studies have observed CaCO_3_ formation in NaOH-OAE systems, but only when pH exceeds 9 for extended periods.^324^ Rapid dilution can suppress this feedback by reducing Ω below critical thresholds.^263^ Current research continues to refine the thresholds and induction times for CaCO_3_ formation.^272,274,320,325,326^

At the global scale, although ocean acidification is expected to suppress biological calcification, its net effect on whole-ocean TA accumulation appears minimal.^327^ Observed relationships between surface-ocean CaCO_3_ inventories and carbonate system parameters are weak and spatially variable,^275^ leaving the large-scale coupling between OAE and CaCO_3_ cycling poorly constrained. To resolve these uncertainties and evaluate the broader implications of OAE for the ocean carbon system, investigations should leverage natural systems exhibiting strong TA gradients^329^ in combination with coordinated large-scale observations, including repeat hydrography, satellite remote sensing, and targeted process studies.

### 4.3 CO_2_ Uptake Efficiency by Alkalinity-Enhanced Seawater

Adding alkalinity to seawater enhances atmospheric CO_2_ uptake, with the CO_2_ flux into seawater (F_CO2_) proportional to the TA flux (F_TA_) through an efficiency factor η_CO2_:

$$F_{\mathrm{CO}2}=\eta_{\mathrm{CO}2}\cdot F_{\mathrm{TA}}$$ 40

This relationship, adapted from Geerts et al.,^277^ expresses both fluxes in mol m^–2^ time^–1^. In a closed system, η_CO2_ is thermodynamically defined by *T*, *S*, and carbonate equilibria.^63^ It is equivalent to the inverse of the isocapnic quotient (i.e., ΔTA/ΔDIC), which represents the change in TA required to maintain constant *p*CO_2_ per unit change in DIC^328^ as

ηCO2=ΔDIC/ΔTA=K1[CO2*]([H+]+2K2)(KB+[H+])2/[(K1[CO2*][H+]+4K1K2[CO2*]+KW[H+]+[H+]3)(KB+[H+])2+KBTB[H+]3] 41

It should be noted that this equation only considers contributions from carbonate and borate species to TA. At the sea surface conditions (TA = 2150 μmol kg^–1^, DIC = 1900 μmol kg^–1^), η_CO2_ typically approximates 0.83 and is largely insensitive to *T* or *S* (Figure 10a), but highly sensitive to TA:DIC ratio (Figure 10b). Notably, η_CO2_ reaches up to 0.95 in waters with low TA:DIC, indicating that smaller alkalinity additions in such waters are more efficient for CDR (e.g., moving vertically in Figure 10b).

**Figure 10:**
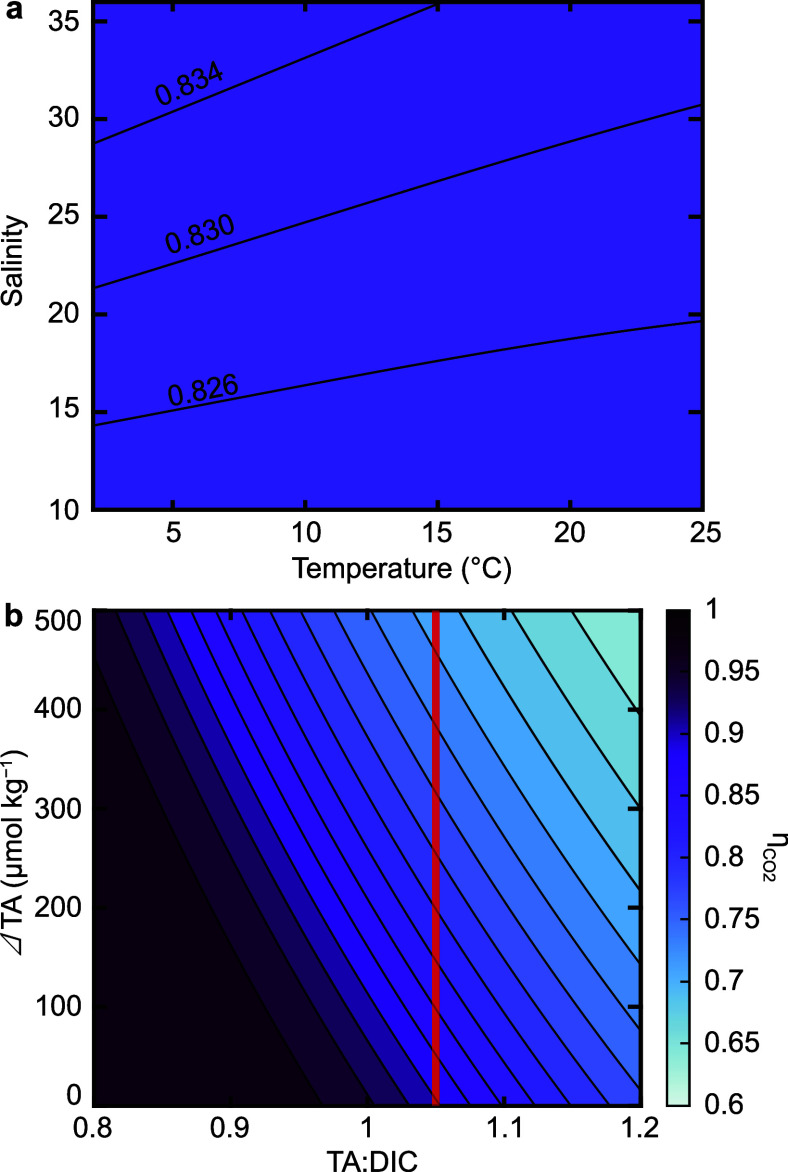
(**a**) Sensitivity of η_CO2_ to *T* and *S* at constant TA (2150 μmol kg^–1^) and DIC (1900 μmol kg^–1^). Contours demonstrate changes in the third decimal place. (**b**) Sensitivity to baseline TA:DIC and alkalinity enhancement (ΔTA), calculated at *S* = 33 and *T* = 15 °C. Mean-ocean TA:DIC (1.05) is shown as the vertical red line.

For net CDR via OAE, the air–sea CO_2_ gradient must exceed the natural background. If generated alkalinity is diluted or subducted before equilibrating with atmospheric CO_2_, the DIC gain is reduced, and η_CO2_ declines (see eq 41),^263,329^ decoupling practical efficiency from the thermodynamic ideal. To illustrate this, a box model was applied with a surface ocean (surf) receiving a TA flux and mixing with a deeper background reservoir (bkgd) isolated from the atmosphere (Figure 11f). The time-dependent changes in TA and DIC are

$$\mathrm{dTA}/\mathrm{dt}=F_{\mathrm{TA}}-k_{\mathrm{mix}}\left({\mathrm{TA}}_{\mathrm{surf}}-{\mathrm{TA}}_{\mathrm{bkgd}}\right)$$ 42

$$\mathrm{dDIC}/\mathrm{dt}=F_{\mathrm{CO}2}-k_{\mathrm{mix}}\left({\mathrm{DIC}}_{\mathrm{surf}}-{\mathrm{DIC}}_{\mathrm{bkgd}}\right)$$ 43

**Figure 11:**
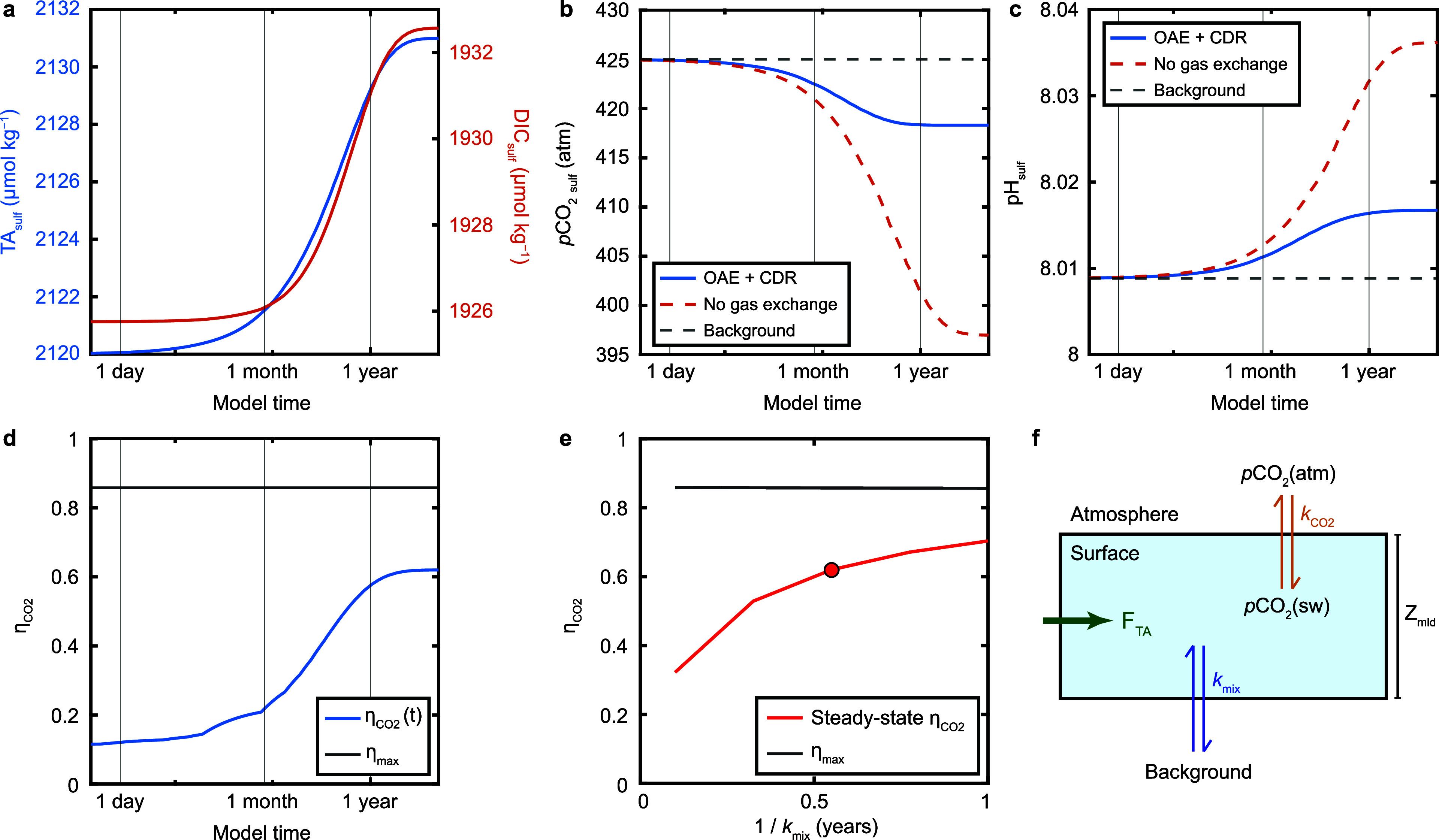
Results from the surface ocean box model using mixed layer depth of 75 m, *k*_CO2_ of 0.005 h^–1^, and *k*_mix_ ranging from 0.001–0.0001 h^–1^. Panels (**a**–**d**) show results from the experiment run with *k*_mix_ = 0.0021 y^–1^. (**a**) Time-evolution of surface ocean TA (blue curve) and DIC (red curve). Note that the *x*-axis is on a log-scale. (**b**) Time-evolution of surface ocean *p*CO_2_ (blue curve). For reference, the background value (black dashed line) is shown, as well as a model run with gas exchange turned off (red dashed curve). (**c**) Time-evolution of surface ocean pH and (**d**) Time-evolution of η_CO2_, with the maximum theoretical η_CO2_ shown in black. (**e**) steady-state η_CO2_ as a function of the surface-deep mixing time scale (*k*_*mix*_). The value for *k*_mix_ used for panels (**a**–**d**) is shown as a red circle in panel (**e**). (**f**) Schematic of the box model geometry, with key fluxes and parameters shown.

Here, *k*_mix_ is the mixing rate, and the gradients represent exchange between surface and background waters. At steady state, η_CO2_ is given by the ratio of fluxes: η_CO2_ = F_CO2_/F_TA_. The box model (Figure 11f) was initialized with a constant F_TA_ (0.0023 μmol kg^–1^ hr^–1^, or 20 μmol kg^–1^ yr^–1^) and various *k*_mix_ values (0.0001–0.001 h^–1^) to examine how mixing influences η_CO2_. DIC increases less quickly than TA due to slower air–sea CO_2_ exchange (*k*_CO2_ = 0.005 h^–1^; piston velocity = 0.37 m hr^–1^; mixed layer depth = 75 m). Figures 11b and 11c show that in the absence of gas exchange, *p*CO_2_ falls and pH rises (red dashed lines). When gas exchange is included, *p*CO_2_ decreases less and pH increases less, stabilizing at new steady states (blue lines). This persistent *p*CO_2_ gradient supports continued CO_2_ uptake, leading η_CO2_ to rise over time and plateau below its theoretical maximum (Figure 11d). Figure 11e highlights that even modest mixing (e.g., *k*_mix_ 43 times smaller than *k*_CO2_) reduces η_CO2_. This is because CO_2_ comprises only a small portion of total DIC,^330^ meaning air–sea CO_2_ exchange is inherently slow. This lag is governed by the CO_2_ relaxation time scale (τ_CO2_)^330,331^ as

$$\tau_{\mathrm{CO}2}=\left(\mathrm{DIC}/\left[{{\mathrm{CO}}_{2}}^{*}\right]\right)/\left(k_{\mathrm{CO}2}\cdot \mathrm{Revelle factor}\right)$$ 44

where the DIC/[CO_2_*] ratio indicates the ionization fraction and the Revelle factor means the sensitivity of surface *f*CO_2_ to changes in DIC (see eq 17). In the model, the CO_2_ relaxation time scale is about 0.26 years, much longer than the gas exchange time scale alone and comparable to mixing time scales (Figure 11e).

This model illustrates that vertical dilution and ocean circulation critically affect OAE efficiency. CO_2_ uptake occurs over extended time scales (months to years), making it essential to incorporate larger-scale models when monitoring OAE-driven carbon removal.^332^ Regional ocean circulation also influences efficiency. In the North Atlantic, rapid subduction limits CO_2_ equilibration, yielding low η_CO2_.^263^ In contrast, subtropical regions may retain alkalinity-rich waters near the surface, allowing partial re-equilibration through lateral and vertical mixing and leading to higher η_CO2_.^329^ Regional ocean models^333^ suggest that shallow coastal zones may be advantageous for OAE due to prolonged surface residence times. However, accurate model-based assessments require robust biogeochemical modules, as TA and DIC are affected by biological processes and feedbacks.^334^ Further ocean model development is needed in parametrizing key processes such as CaCO_3_-associated processes and sediment interactions.^335^

### 4.4 Monitoring, Reporting, and Verification

A central challenge of OAE is quantifying the amount of atmospheric CO_2_ removed as a result of the intervention. This carbon accounting process, known as monitoring, reporting, and verification (MRV), relies on establishing *additionality*—defined as carbon uptake that exceeds what would have occurred without the intervention.^336^ Establishing additionality requires defining a counterfactual baseline in which no TA addition takes place.

MRV for OAE is complicated by the natural dynamic alkalinity cycle, which may be altered by exogenous alkalinity inputs.^275,336^ Moreover, model simulations indicate that some portion of CDR occurs over large ocean areas, often driven by subtle or unmeasurable surface CO_2_ gradients.^263,329,333,337^ Consequently, early OAE carbon credit protocols rely on model-based CDR estimates, using simulations with and without alkalinity addition as counterfactual baselines.^338^ However, persistent discrepancies between model estimates and observations of global oceanic carbon uptake highlight the need for coordinated advances in both observational capacities and modeling approaches, as well as transparent and accessible data.^339^ Such efforts are essential not only for quantifying atmospheric CO_2_ uptake but also for verifying CDR achieved through OAE.^332,340^

#### 4.4.1 Monitoring CO_2_ Uptake

MRV practices for OAE can build upon decades of oceanographic process studies (Figure 12).^332,341^ MRV will rely on multiparameter carbonate chemistry measurements, including shipboard bottle samples (via Niskin rosettes) and continuous surface data from underway systems.^342^ To attribute CO_2_ uptake to OAE interventions, especially in small-scale field trials, it is strongly recommended to use water tracers. These tracers help distinguish physical processes like mixing, dilution, and gas exchange from biogeochemical changes in the carbonate system.^340,341^ Such tracers may become cost-prohibitive or logistically infeasible as projects scale up or transition to routine operations.^341,342^ Nevertheless, they are critical at early stages to ground-truthing dilution and dispersion models, especially in the vicinity of the intervention site.

**Figure 12:**
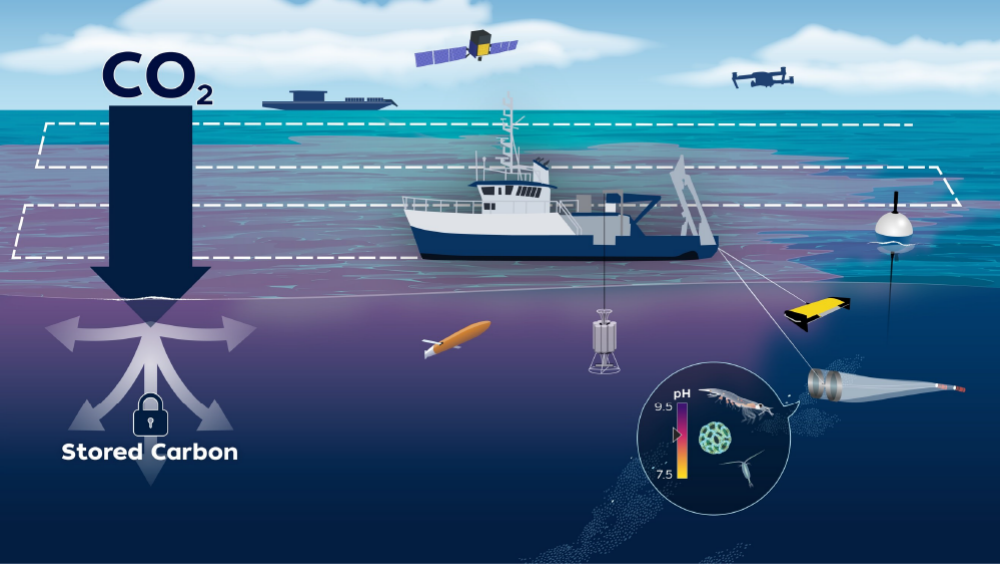
An illustration of in-water monitoring approaches for OAE, showing vessel deployments of a rosette sampler, a towed sensor package, and a plankton net. Other measurement platforms include autonomous gliders, remote sensing via drones and satellite imagery, and drifting buoys equipped with sensors. An OAE intervention can be labeled with a water tracer (e.g., the pink colored water), and can be sampled systematically (e.g., along the dashed line), along with the background seawater, to measure OAE efficiency and environmental impacts. Illustration by Eric Taylor ©Woods Hole Oceanographic Institution.

Each carbonate system parameter presents unique trade-offs in terms of measurement precision, resolution, and deployability.^27^ Recent advances in TA and pH sensors have improved in situ capability and resolution across platforms, including autonomous vehicles.^343,344^ The OAE-driven CO_2_ uptake is reflected in changes in TA, DIC, *p*CO_2_, and pH. Among these, only DIC accumulation—indicating actual carbon storage—occurs when gas exchange accompanies OAE, while *p*CO_2_ and pH respond to both processes (Figure 13).^342^ However, high-precision DIC measurement remains challenging, making TA in combination with *p*CO_2_ or pH the most practical MRV targets.^342,343^ These can be obtained from shipboard surveys or autonomous platforms like gliders, though it is essential to match sampling resolution with the spatial and temporal scales of the OAE deployment.^343,345^

**Figure 13:**
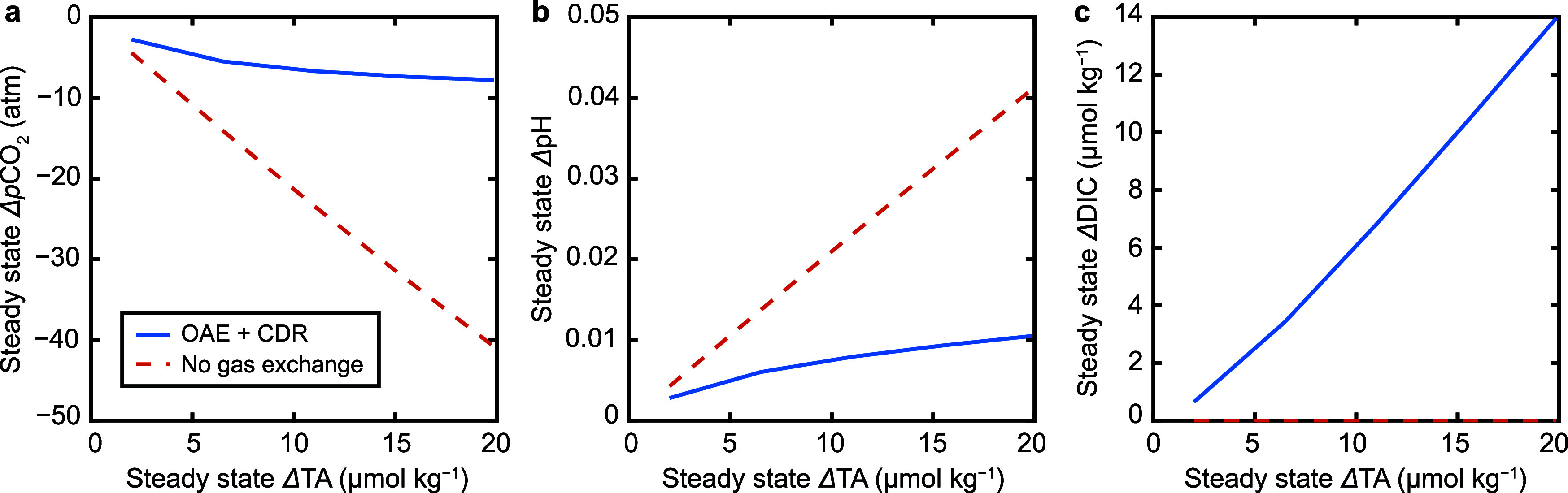
Steady-state box model output from the model presented in Section 4.3, shown for the configuration identified by the red dot in Figure 11e. Changes relative to the baseline in (**a**) *p*CO_2_, (**b**) pH, and (**c**) DIC, are shown as a function of TA enhancement. Model runs in which gas exchange is turned off (red dashed lines) are compared to model runs with gas exchange (blue lines), demonstrating the strong signals in *p*CO_2_ and pH driven by CO_2_ uptake into the surface ocean. DIC only accumulates if gas exchange is turned on.

Monitoring strategies must also adapt to the scale and type of OAE intervention (Figure 8). For example, sediment-based applications^316^ require different metrics than outfall-based^294^ or open-ocean ship-based methods.^323,342^ Typically, monitoring is divided into near-field and far-field regimes.^332,335,338^ The near-field—close to the intervention site—shows the strongest OAE signals in TA, pH, turbidity, and potential biological effects from elevated pH, trace metals, or particulates. Although most long-term CO_2_ uptake may occur in the far-field over broader oceanic regions with subtle TA and *p*CO_2_ gradients,^263,329^ the near-field offers the clearest signal for calibrating fluxes and developing parametrizations.^332,341,342^ Near-field monitoring must account for regional variability, especially in complex coastal and estuarine systems.^346^ In data-limited regions, regression and machine learning techniques can help interpolate sparse measurements.^347^ In contrast, the far-field will exhibit weaker TA signatures due to dilution but may accumulate more CO_2_ over time through continued gas exchange.

Numerical modeling will play a central role in MRV.^335^ Models must capture both near- and far-field processes and include modules for sediment dynamics, mixing, and dilution.^323,342,348^ Regional and global frameworks are needed to quantify carbon removal over broad spatial and temporal domains. Near-field models must consider alkalinity production and delivery mechanisms (see Section 4.2), while far-field models should incorporate large-scale biogeochemical feedbacks, such as calcification.^275,327^

All models should be validated against observations collected at compatible scales.^2^ As OAE scales up, its effects will be increasingly embedded within the global carbon cycle, making sustained, high-quality observations essential for improving process understanding, refining estimates of additionality, and enhancing global carbon accounting. Critically, earth-system feedbacks and the whole-ocean responses may mask direct signals of atmospheric CO_2_ drawdown attributable to OAE, further complicating MRV efforts.^349^ Open and transparent data sharing, supported by standardized protocols currently under development,^350^ will be crucial for building trust and ensuring the robustness of MRV frameworks.^351^

#### 4.4.2 Monitoring Environmental Impacts

OAE may mitigate ocean acidification by lowering proton levels, thereby alleviating pH-related stress on marine organisms.^267,352^ However, excessive pH increases can negatively affect sensitive life stages—such as larvae, juveniles, and nonmotile organisms (e.g., phytoplankton, eggs)—that cannot actively escape altered conditions.^353–355^ While elevated bicarbonate levels, which form as added alkalinity equilibrates with atmospheric CO_2_, are generally well tolerated,^356^ maintaining pH within ecologically safe limits during and after OAE is critical. Most experimental studies have focused on phytoplankton, typically reporting neutral or minor responses to added alkalinity.^273,357–359^ Recent work has extended to zooplankton, including copepods and their grazing behaviors.^354,360^ However, broader studies across multiple trophic levels—and on interactions within food webs—are needed to fully understand OAE’s ecological effects, especially as OAE scales up.

In the U.S., the Environmental Protection Agency^361^ recommends maintaining coastal water pH between 6.5 and 8.5. Given the widespread availability of pH sensors and its regulatory relevance, in addition to its sensitivity to both OAE and CO_2_ uptake, pH is a practical and effective metric for environmental monitoring of OAE, more so than TA. Figure 19 illustrates how OAE-induced changes in pH and Ω_calcite_ for calcite vary with *T* and *S*. The TA increment needed to reach pH 8.5 rises in warmer waters, reflecting pH’s inverse sensitivity to *T* (Figure 14a). Meanwhile, Ω_calcite_ slightly increases with *T*, as calcite becomes less soluble (Figure 14b). The conservative threshold for spontaneous calcite precipitation is only approached under warm, high pH conditions where both pH and Ω_calcite_ thresholds align (red dotted lines in Figures 14b and d). Thus, maintaining pH within accepted ranges protects marine life and minimizes the risk of unintended CaCO_3_ precipitation during OAE deployments.

**Figure 14:**
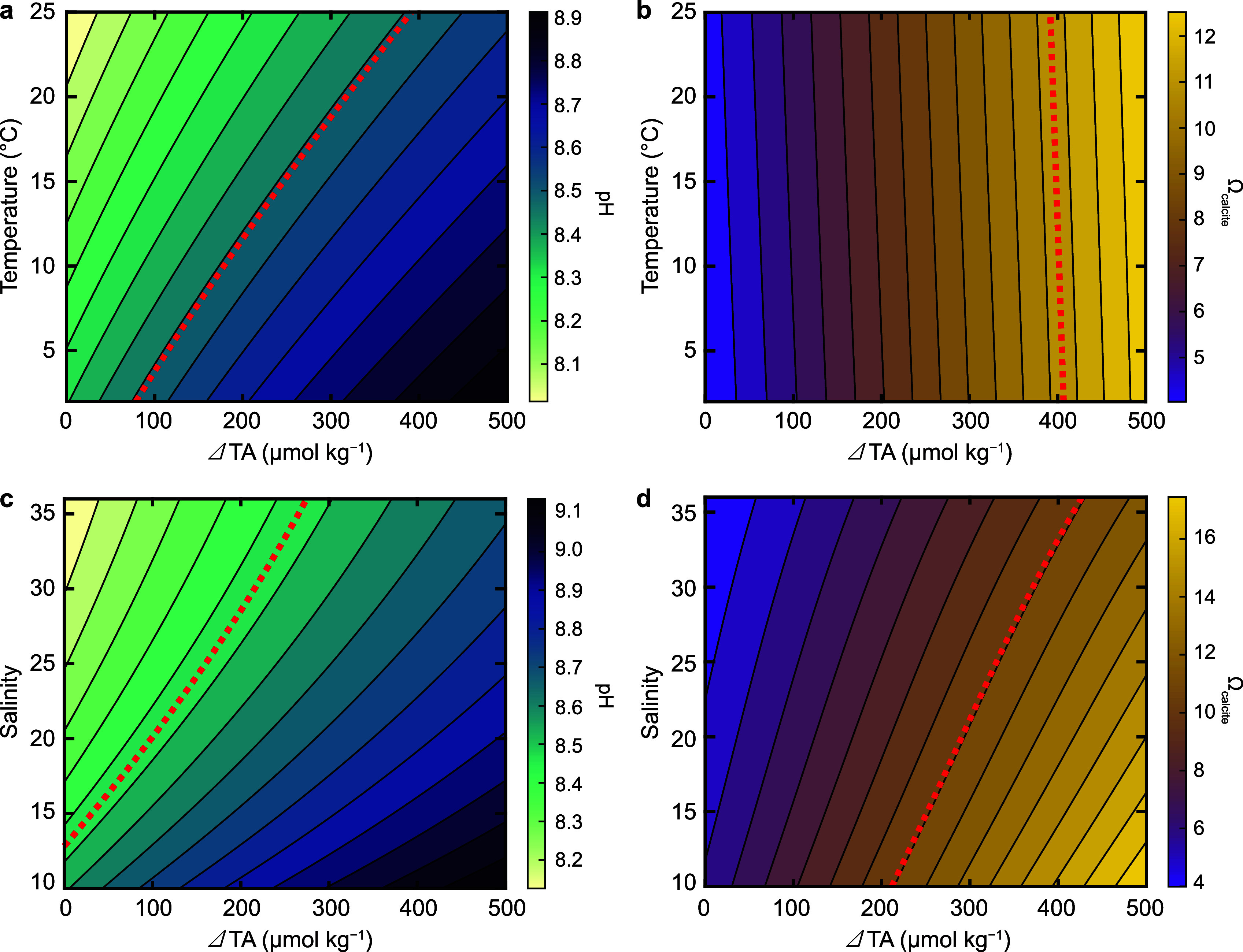
Interaction of (**a**, **b**) temperature (*T*) and (**c**, **d**) salinity (*S*) with OAE on the marine carbonate system, calculated using CO2SYS with a fixed baseline DIC of 1900 μmol kg^–1^, TA of 2150 μmol kg^–1^. Influence of *T* on (**a**) pH and (b) Ω_calcite_ are calculated at a fixed *S* of 33. Influence of *S* on (**c**) pH and (**d**) Ω_calcite_ are calculated at a fixed *T* of 15 °C. The red dotted lines indicate the pH 8.5 threshold for panels (**a**) and (**c**) and the spontaneous precipitation threshold as Ω_calcite_ of 11.1 from ref 272 for (**b**) and (**d**). The thresholds of ref 325 are too high to display on this plot.

In addition to pH and carbonate chemistry, turbidity from solid slurries, suspensions, or sediment amendments is a key environmental concern for OAE. Material impurities, particularly in olivine, may introduce trace metals or other harmful constituents.^17,287^ Increased marine activity may also conflict with other ocean uses,^362^ and heightened vessel traffic raises the risk of marine mammals collisions. Additionally, sediment-based applications risk disturbing sensitive habitats such as spawning or nursery grounds. These potential impacts underscore the importance of marine spatial planning to minimize ecological disruption and avoid overlapping stressors. To ensure comprehensive assessment, ecosystem and fisheries models must incorporate both biogeochemical and biological effects of OAE interventions.

#### 4.4.3 Life Cycle Assessment and Net CO_2_ Removal Accounting

To quantify net CDR from OAE, all CO_2_ emissions associated with the intervention must be accounted for. Accordingly, carbon credits should be issued only for net CDR:

$${\mathrm{CDR}}_{\mathrm{net}}={\mathrm{CDR}}_{\mathrm{gross}}-{\mathrm{CO}}_{2,\mathrm{emit}}$$ 45

This requires a comprehensive life cycle analysis that includes all emissions, including those from material production, transport, deployment, and monitoring activities. While life cycle analysis frameworks exist in the literature, studies specific to OAE are still limited.^17,289^ A recent case study in Halifax Harbor involving the dispersal of Mg­(OH)_2_ demonstrated measurable net CDR both at the point of discharge and over the mineral’s dissolution lifetime.^294,363^ Approximately 278 tonnes of Mg­(OH)_2_ were added to seawater, yielding a theoretical maximum of 9.73 Mmol alkalinity (Table 2). Assuming complete equilibration with atmospheric CO_2_ and η_CO2_ of 0.83, this corresponds to 355 tonnes of gross CDR. After accounting for associated emissions, the net CDR credited was 138 tonnes—indicating that 217 tonnes of CO_2_ were emitted during the process. This implies that the current system operates at an efficiency of less than 50%, implying that to meet a specific CDR target, at least twice the theoretical amount of alkaline material would need to be applied. Whether economies of scale, or technological innovation, can significantly improve this efficiency over time remains an open question.

## 5 Macroalgae-Based Strategies for Carbon Storage

### 5.1 Overview

Macroalgae are the most productive autotrophs in coastal ecosystems, with a global net primary production (NPP) of approximately 1.3 Pg C yr^–1^ distributed across 6–7 million km^2^,^364^ exceeding the combined NPP of blue carbon ecosystems (BCEs) such as mangroves, salt marshes, and seagrasses.^365–367^ Owing to this high productivity and broad spatial distribution, macroalgae are increasingly recognized as having potential for marine carbon dioxide removal (mCDR). Among macroalgae, canopy-forming kelps and *Sargassum* typically exhibit high efficiencies in light utilization and DIC assimilation, owing to their pigment composition and carbon-concentrating mechanisms.^367–369^

Through rapid growth and CO_2_ uptake, macroalgae can substantially lower surface *p*CO_2_, enhancing CO_2_ flux into the ocean. This mechanism parallels the phytoplankton-driven biological (soft-tissue) pump (Section 3.1.2), but with higher carbon fixation per unit nutrient uptake, yielding greater photosynthetic efficiency.^370,371^ However, high primary productivity does not necessarily translate into durable carbon storage.^372^ A large fraction of fixed carbon is released through tissue shedding, exudation of particulate and dissolved organic matter, and decomposition during senescence.^373–377^ Only a limited portion contributes to long-term storage—on time scales exceeding 100 years relevant to CDR—via (1) particulate organic carbon export and burial in deep-sea sediments,^378–380^ (2) transformation into refractory dissolved organic carbon resistant to microbial degradation,^379,381^ or (3) production of bicarbonate ion, which enhances seawater alkalinity and carbon storage capacity^382,383^ (Figure 15; see Section 5.2).

**Figure 15:**
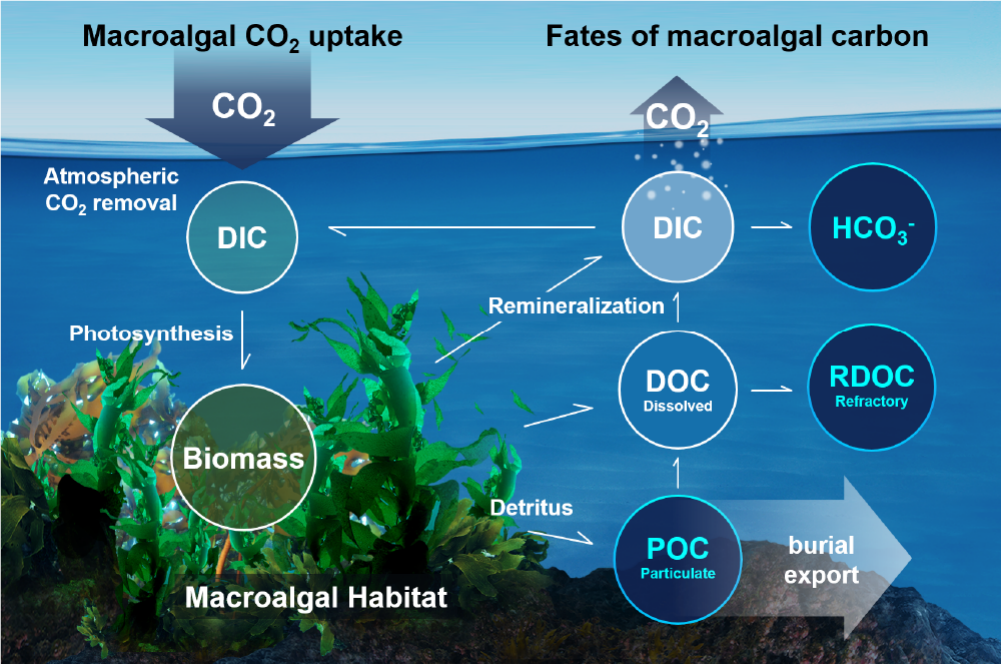
Pathways of atmospheric CO_2_ uptake and storage mediated by macroalgae.

Beyond natural pathways, a range of intervention have been proposed to augment macrolagae-based CDR.^384–386^ Coastal restoration seeks to recover degraded kelp forests and their CDR capacity, whereas large-scale aquaculture and offshore afforestation aim to establish new macroalgal habitats beyond historical ranges (Figure 16). These can be coupled with biomass harvesting and deliberate sinking to promote deep-ocean carbon isolation. Harvested biomass may also serve as feedstock for durable carbon products such as biochar, bioplastics, biofuels, or animal feed. Despite their potential, the net climate benefits of these interventions remain uncertain due to ecological feedbacks and operational energy demands.^387–391^

**Figure 16:**
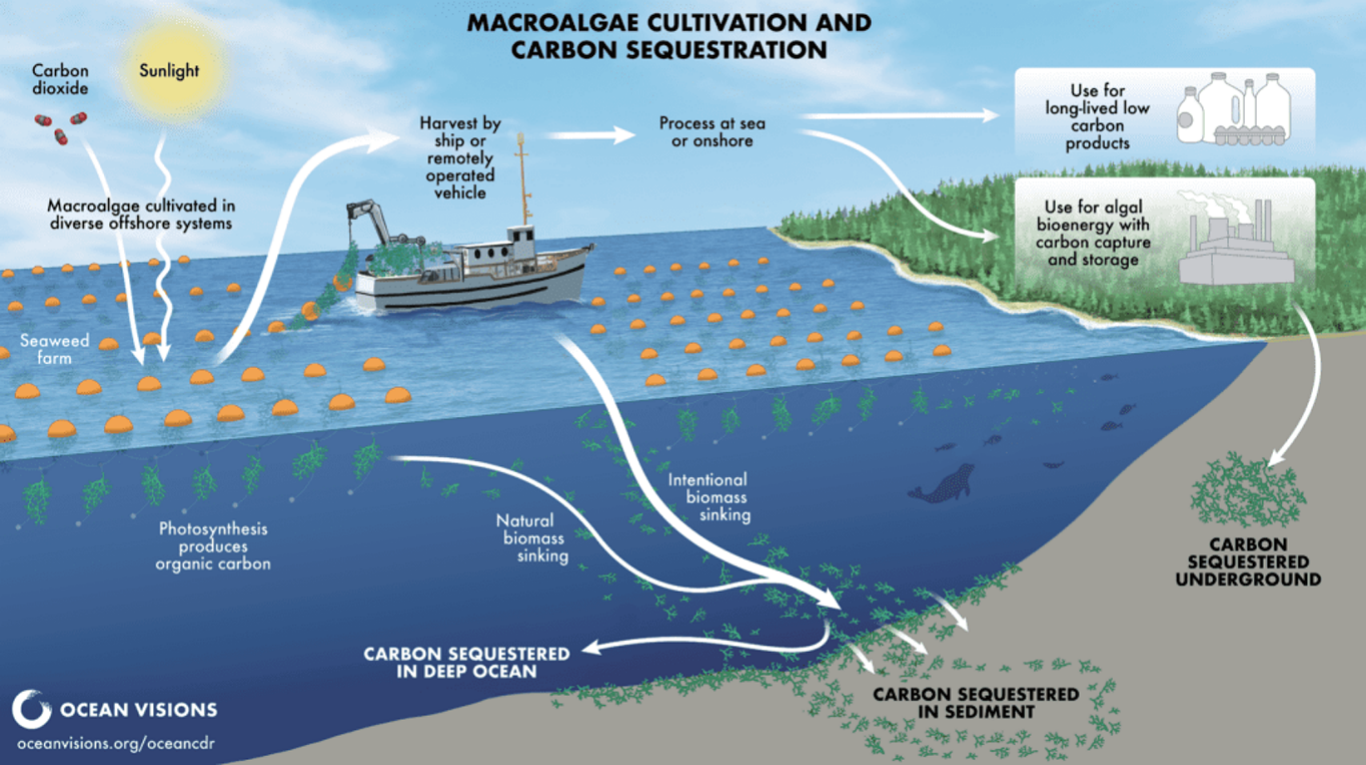
Illustration of macroalgae cultivation and carbon storage. Reproduced from Ocean Visions, Copyright 2023 Ocean Visions under Creative Commons CC BY-NC-ND license https://creativecommons.org/licenses/by-nc-nd/4.0/.

This section reviews macroalgal contributions to mCDR through three complementary perspectives: (1) natural storage pathways (Section 5.2); (2) engineered interventions and their associated biogeochemical feedbacks (Section 5.3); and (3) methodological challenges and constraints related to monitoring, reporting, and verification (Section 5.4).

### 5.2 Macroalgal Carbon Storage Pathways

Quantifying the contribution of macroalgae to long-term carbon storage remains challenging, primarily because most macroalgal assemblages grow on rocky substrates where direct in situ burial of organic carbon is negligible.^366,379,392^ Instead, a significant fraction of macroalgal production—estimated at ∼ 44% on average, though highly variable among regions—is exported from source habitats to adjacent environments, a proportion substantially greater than that of most other marine autotrophs.^378,379,393^ Understanding the fate and transformation of this exported material is therefore fundamental to assessing macroalgae-based carbon storage. Key pathways include its transport to deep ocean and burial in depositional sediments, conversion into refractory dissolved organic carbon, and transformation into bicarbonate ion. Collectively, these processes determine the proportion of macroalgal carbon that is isolated on climatically relevant time scales.

#### 5.2.1 Particulate Organic Carbon Export and Burial

Macroalgae generate particulate organic carbon (POC) throughout their life span, from the release of spores and gametes to the detachment of thalli during senescence, grazing, epibiont damage, and hydrodynamic disturbance.^378^ The resulting POC spans a continuum from fine detrital particles to large drifting thalli, with detrital release accounting for ∼ 80% of NPP in kelp forests.^378^

Most POC is remineralized within source habitats through microbial decomposition and grazing, and only about 0.4% of NPP is buried locally.^379,393^ Nevertheless, a notable fraction is advected offshore, where its fate depends on species composition, hydrodynamics, seasonal conditions, and depth gradients.^394,395^ Coastal macroalgal forests may export up to 15% of global NPP (Figure 17a). Of the exported detritus, less than 20% transported below 200 m persists for over a century, whereas roughly 50–80% of carbon reaching depths greater than 1,000 m remains stored on centennial timescales (Figure 17b).^397^ Approximately 0.9% of NPP is ultimately buried in continental shelves, while ∼ 2.3% reaches the deep ocean, where it is effectively isolated from atmospheric exchange on centennial to millennial time scales.^379,396^

**Figure 17:**
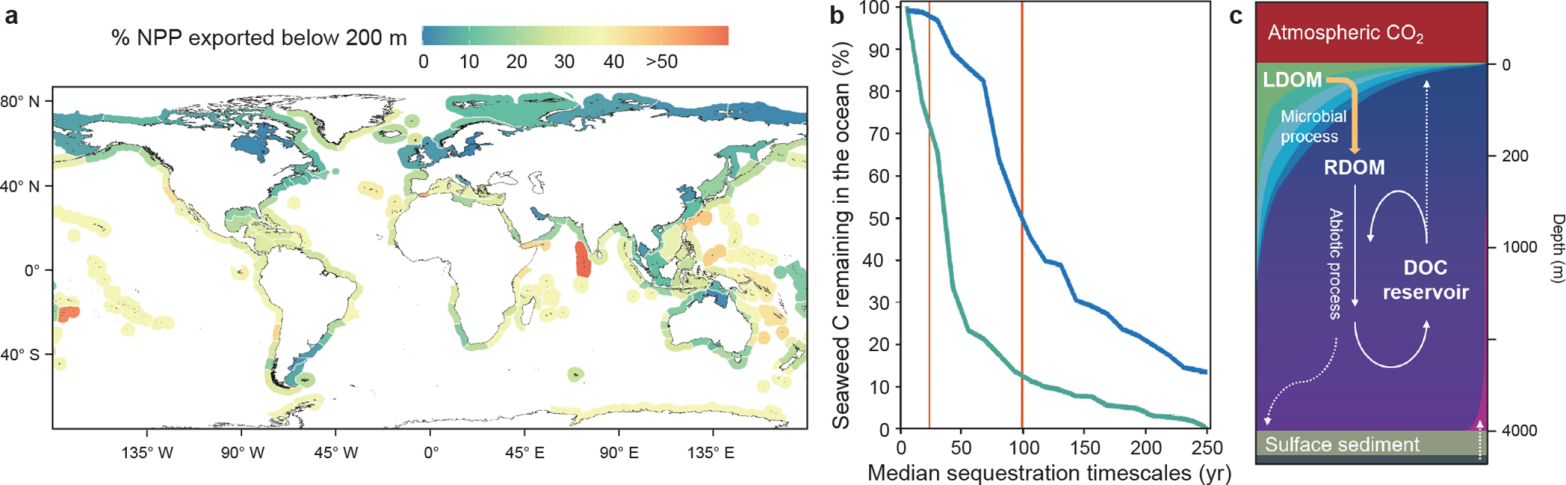
(**a**) Average percentage of macroalgal NPP exported beyond the continental shelves as POC. (**b**) Cumulative macroalgal carbon exported beyond the continental shelves across different storage time scales. (**c**) Transformation of labile DOM into refractory DOM via microbial processes. Panels (**a**) and (**b**) reproduced with permission from ref 397. Copyright 2024 Springer Nature. (**c**) Adapted with permission from ref 381. Copyright 2010 Springer Nature.

Collectively, these pathways indicate that approximately 3.6% of global macroalgal NPP may be stored as POC within nearshore sediments, continental shelves, and deep-ocean basins.^379^ However, quantifying these fluxes remains difficult due to methodological challenges in tracing and attributing macroalgal-derived carbon in depositional environments (see Section 5.4).

#### 5.2.2 Refractory Dissolved Organic Carbon Storage

The global marine dissolved organic carbon (DOC) reservoir, estimated at ∼ 700 Pg C, is comparable in magnitude to the atmospheric carbon pool.^2^ Although constituting only ∼ 18% of the total oceanic carbon inventory, it exceeds the global marine biomass by more than 2 orders of magnitude,^2,398,399^ emphasizing its significance as a long-term carbon reservoir. While most marine DOC originates from planktonic production, macroalgae contribute substantially—up to 20% of coastal DOC inputs.^400^

Macroalgae release DOC through both active and passive processes, including metabolic overflow, herbivore deterrence, diffusion, and osmotic stress responses.^401,402^ Additionally, particulate detritus shed during senescence can be rapidly solubilized into DOC, although conversion efficiencies vary across systems.^403,404^ Collectively, DOC release accounts for 13–35% of macroalgal NPP, highlighting its significance in coastal carbon cycling.^379,402,405,406^

Most DOC produced by macroalgae is rapidly remineralized near its source, typically within algal beds or the surface mixed layer. A small fraction, however, is exported offshore with global estimates suggesting that up to 8% of macroalgal NPP may be transported below the mixed layer.^379^ Portions of exported DOC contribute to oceanic carbon reservoirs consisting of refractory RDOC—carbon-rich, structurally complex molecules formed through microbial processes (Figure 17c).^381,407^ RDOC is resistant to microbial degradation and can persists in the deep ocean for millennial time scales.^407–409^ The proportion of macroalgal DOC converted to RDOC varies widely (25–60%), influenced by microbial community structure, temperature, and nutrient availability.^406,410–413^ Despite this variability, RDOC formation constitutes one of the most persistent and durable pathways of macroalgal carbon storage.^414^

#### 5.2.3 Bicarbonate Ion Formation under Oxygenated Conditions

Beyond organic pathways, macroalgal degradation can contribute to inorganic carbon storage through the formation of bicarbonate ion. During oxic remineralization, organic carbon is converted to CO_2_,^415^ which readily exchanges with the atmosphere and thus provides no CDR benefit. In contrast, anoxic microbial processes such as denitrification and sulfate reduction transfer negative charge from nitrate or sulfate, to carbon generating bicarbonate ion and increasing seawater TA.^416^ Because bicarbonate ion is chemically stable and resistant to outgassing, this pathway represents a potentially durable form of inorganic carbon storage.

Traditionally, alkalinity generation from organic matter degradation was considered to occur primarily in anoxic environments, where oxygen depletion favors such redox reactions.^412,417,418^ Laboratory studies indicate that up to ∼ 10% of sunken macroalgal carbon can be converted to bicarbonate ion under these conditions,^412^ contributing to long-term inorganic carbon storage in coastal sediments.

Recent studies indicate that sulfate reduction can occur in oxygenated water columns. Anoxic microenvironments—such as within macroalgal tissues, detrital aggregates, or biofilms—can sustain sulfate-reducing microbial activity despite oxic surroundings.^259,419–421^

$$2\left({\mathrm{CH}}_{2}O\right)+{{\mathrm{SO}}_{4}}^{2-}\to 2{{\mathrm{HCO}}_{3}}^{-}+H_{2}S$$ 46

Reduction of one mole of sulfate generates two moles of alkalinity through bicarbonate production. The accompanying hydrogen sulfide (H_2_S) may either escape to the atmosphere or undergo oxidation within the water column. Atmospheric release does not alter the alkalinity gain, whereas oxidation can partially offset it. Despite this potencial offset, field observations consistently show a net increase in total alkalinity during macroalgal decomposition under oxygenated conditions (Figure 18).^382,383^

**Figure 18:**
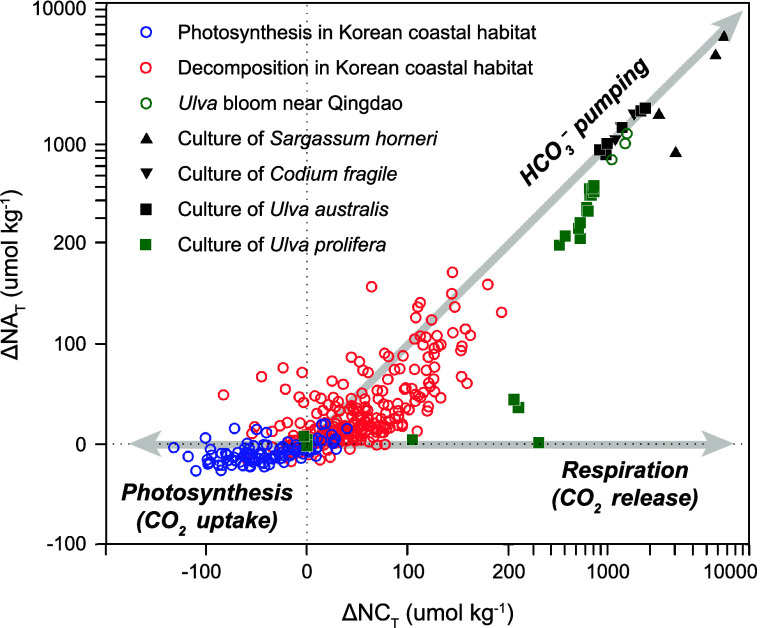
Changes in salinity-normalized TA and DIC after correction for nutrient effects, organic alkalinity, air–sea CO_2_ exchanges, associated with macroalgal biogeochemical processes. Field observations (open circles) and culture experiments (filled symbols) are shown on a conceptual background with gray arrows indicating the directions of photosynthesis (leftward), respiration (rightward), and HCO_3_^–^ generation via sulfate reduction (upward at 45°). Adapted with permission from ref 383. Copyright 2025 John Wiley and Sons.

Despite its potential significance, this pathway remains poorly constrained, and its global contribution to inorganic carbon storage is not yet quantified. Targeted field observations and mechanistic experiments are needed to resolve its spatial distribution, identify key environmental controls, and incorporate it into comprehensive assessments of macroalgal-based CDR.

### 5.3 Feedback to Macroalgae-Based Interventions

To enhance carbon storage beyond the natural capacity of macroalgal forests, several intervention strategies have been proposed, including large-scale coastal aquaculture, open-ocean afforestation, and the deliberate sinking of harvested biomass into the deep ocean. All such approaches ultimately depend on the storage pathways outlined in Section 5.2.

Observational and modeling studies indicate that organic carbon deposition beneath macroalgal farm occurs at rates comparable to the lower end of estimates for other BCEs.^422^ Achieving macroalgal biomass production on the order of 1 Pg C yr^–1^ would require cultivation across approximately 1 million km^2^ of highly productive exclusive economic zones.^423^ However, both biomass yield and the permanence of storage are constrained by a suit of biogeochemical feedbacks—including nutrient limitation, CO_2_ uptake offsets, and ecosystem perturbations—that can substantially reduce net efficiency (Figure 19a). The additionality of macroalgae-based CDR therefore depends on recognizing and mitigating these feedback mechanisms. Optimizing site selection, nutrient recycling, and cultivation practices, while minimizing ecological disruption, will be essential to ensure that enhanced macroalgal production translates into durable, verifiable CDR.

**Figure 19:**
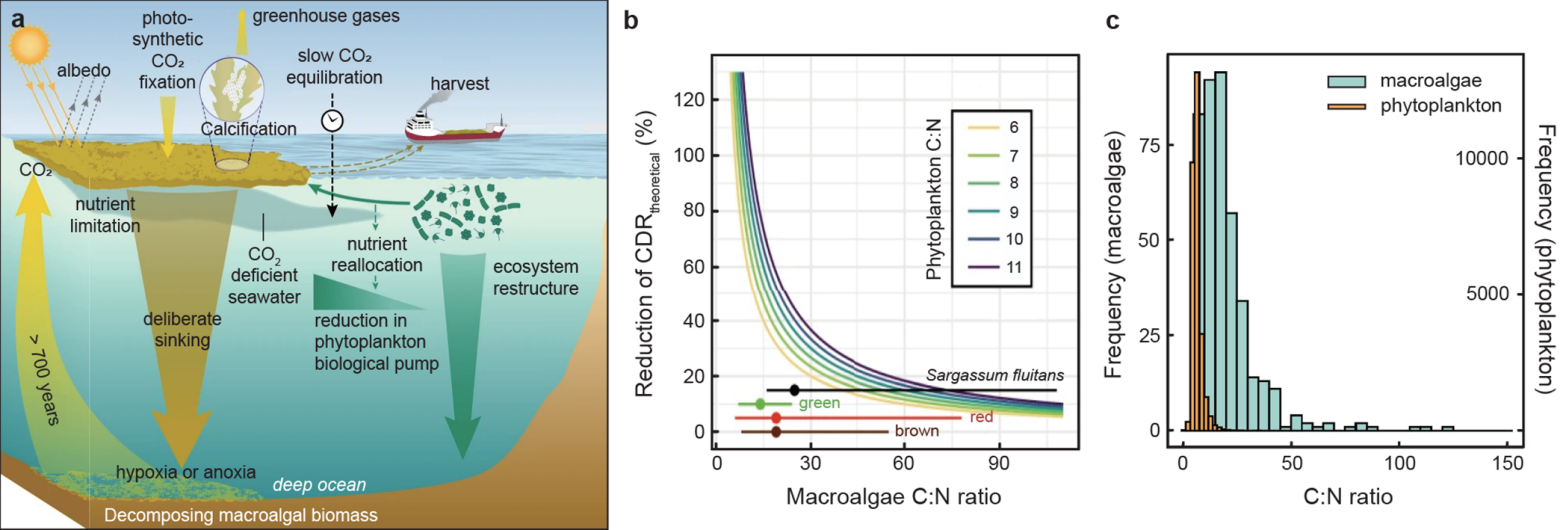
(**a**) Feedback mechanisms associated with macroalgae-based CDR. (**b**) Theoretical CDR potential reduced by nutrient reallocation. Horizontal bars indicate the C:N ratio ranges for *Sargassum*, green, red, and brown macroalgal species, respectively, with symbols representing the mean values for each group. (**c**) Histogram for comparing C:N ratios between macroalgae and phytoplankton. Panels (**a**) and (**b**) adapted from ref 424. Copyright 2021 Springer Nature under Creative Commons CC BY license https://creativecommons.org/licenses/by/4.0/. (**c**) Adapted from ref 370. Copyright 2023 John Wiley and Sons under Creative Commons CC BY license https://creativecommons.org/licenses/by/4.0/.

#### 5.3.1 Bioavailable Carbon Limitation

Macroalgal photosynthesis is generally not constrained by inorganic carbon availability, as many species possess carbon-concentrating mechanisms that facilitate direct utilization of abundant HCO_3_^–^ (1800–2000 μmol kg^–1^), rather than relying solely on the far less available dissolved CO_2_ (10–20 μmol kg^–1^).^425,426^ However, under conditions of intense photosynthetic activity, local pH can exceed 9, shifting dissolved inorganic carbon speciation toward CO_3_^2–^ and thereby reducing the pool of CO_2_ and HCO_3_^–^ accessible for uptake.^426–428^ Experimental studies show that in dense macroalgal stands with restricted water exchange, this shift can induce localized carbon depletion sufficient to suppress photosynthesis.^426,427,429^ While such conditions are rare in well-mixed coastal habitats, they may become significant in aquaculture or open-ocean cultivation deployments, where hydrodynamic exchange is reduced and canopy densities are elevated.

#### 5.3.2 Calcification

Calcification within macroalgal habitats exerts a dual influence on marine carbon cycling. On one hand, burial of CaCO_3_ within algal beds or underlying sediments constitutes a long-term carbon sink.^430–432^ On the other hand, CaCO_3_ precipitation decreases seawater TA and increases surface *p*CO_2_, releasing CO_2_ to the atmosphere—approximately 0.6 mol of CO_2_ for every mole of CaCO_3_ formed.^318^ This release partially counteracts the CDR benefits of macroalgal production.^424,433^

Even when noncalcifying species are cultivated, macroalgal habitats commonly support calcifying organisms such as coralline algae, epiphytic foraminifera and bryozoans, and grazing mollusks that further diminish net CDR.^424,433^ Quantifying the proportion of calcareous material in total biomass—for example, ∼ 9.4% CaCO_3_ by wet weight in *Sargassum*—is therefore essential for assessing the magnitude of this offset and constraining the true CDR potential of macroalgae-based interventions.^424,434,435^

#### 5.3.3 Nutrient Limitation

Macroalgal growth is primarily constrained by the availability of nitrogen and phosphorus, following Liebig’s law of the minimum.^428,436^ In coastal aquaculture systems, intensive nutrient uptake can rapidly deplete ambient nutrient pools, leading to growth suppression.^437^ Unlike natural habitats, where macroalgae benefit from nutrient regeneration by associated invertebrates and microbial communities,^428,438,439^ cultivation systems are more susceptible to nutrient limitation, particularly because repeated biomass harvesting removes nutrients and disrupts local recycling loops.^440,441^ Nutrient scarcity is even more pronounced in open-ocean afforestation, where background nutrient concentrations are inherently low and macroalgal assemblages lack access to remineralized nutrients from benthic sources.^442–444^ Consequently, large-scale cultivation is intrinsically vulnerable to nutrient limitation, which constrains productivity and reduces the overall CDR potential.

In addition to macronutrients, trace-metal availability—especially iron—can also limit macroalgal growth.^445^ While experimental iron supplementation has been shown to enhance productivity,^445–447^ current regulatory restrictions on offshore iron addition limit its practical application.

#### 5.3.4 Nutrient Reallocation

Nutrient limitation affects both macroalgae and phytoplankton, the latter forming the foundation of the biological carbon pump (Section 3.1.2), whose CDR efficiency is tightly governed by regional nutrient availability.^448–451^ Large-scale macroalgal cultivation may therefore divert nutrients from phytoplankton communities to macroalgae, potentially diminishing plankton-driven CDR potential:^424^

$${\mathrm{CDR}}_{\mathrm{theoretical}}={\mathrm{CDR}}_{\mathrm{macroalgae}}-{\mathrm{CDR}}_{\mathrm{plankton}}$$ 47

Modeling studies suggest that such nutrient reallocation could reduce the net CDR efficiency of *Sargassum* blooms by ∼ 30% (Figure 19b).^424,452^

However, macroalgal biomass typically exhibits higher stoichiometric ratios (e.g., C:N) than phytoplankton, fixing 3- to 4-fold more carbon per unit nutrient consumed (Figure 19c),^370^ and potentially achieving more efficient export to the depth.^371^ Under favorable conditions, this could offset or even enhance net CDR relative to phytoplankton productivity.^433^ Nonetheless, these potential gains must be evaluated against reductions in the pre-existing phytoplankton pump, highlighting the necessity of system-level assessments that capture nutrient competition and feedbacks across ecosystem scales.^424,452^

#### 5.3.5 Feedbacks from Nutrient Supplementation

Engineered nutrient-supply strategies have been proposed to alleviate growth limitations in large-scale macroalgal cultivation. Artificial upwelling, which brings nutrient-rich deep water to the surface, can enhance productivity but simultaneously introduce CO_2_-rich waters that promote outgassing and may disrupt local ecosystem. These opposing effects underscore the importance of rigorous site selection and comprehensive environmental impact assessment.^453–455^ Depth-cycling methods, whereby cultivation structures are submerged into nutrient-rich layers at night and returned to the surface during the day, have achieved up to 4-fold increases in biomass yield.^456^ However, nutrient enrichment can lower macroalgal C:N fixation ratios, reducing the amount of carbon fixed per unit nutrient consumed.^370,457^ Optimizing nutrient supplementation therefore requires balancing enhanced growth with the preservation of high stoichiometric efficiency to maximize overall carbon CDR potential.

#### 5.3.6 Environmental Impacts and Broader Concerns

Beyond feedbacks that directly affect CDR efficiency, macroalgae-based CDR strategies pose environmental, ecological, and societal challenges. Large-scale floating *Sargassum* canopies can increase surface albedo and alter local heat budgets, introducing secondary climate feedbacks.^424,443^ Cultivation structures may disrupt hydrodynamics, altering water mixing and circulation patterns.^443,458^ Massive macroalgal blooms have caused significant socio-economic damage,^459^ and their decomposition can release potent greenhouse gases such as CH_4_, N_2_O, and halocarbons, partially offsetting CDR gains.^443,460,461^

Deliberate sinking of harvested biomass concentrates organic matter at depth, consuming oxygen and potentially generating hypoxic or anoxic conditions that degrade benthic ecosystems.^443,444,462–464^ Expanding macroalgal dominance can also restructure food webs, with cascading effects on fisheries and other marine economic activities.^371,443^ Moreover, large-scale deployment poses technical, economic, and ethical challenges.^442–444^ These broader concerns highlight the need to evaluate macroalgae-based CDR approaches not only in terms of their carbon removal potential but also with respect to their ecological impacts, socio-economic trade-offs, and governance implications.

### 5.4 Monitoring, Reporting, and Verification

With growing interest in macroalgae-based CDR, the development of standardized monitoring, reporting, and verification (MRV) frameworks has become essential.^386,465–467^ Robust and transparent quantification of CDR must satisfy three core criteria: (1) CDR detection and its persistence over climatically relevant time scales; (2) attribution of observed changes specifically to macroalgal interventions rather than natural variability (i.e., additionality); and (3) evaluation of ecological side effects and system-wide feedbacks on marine environments.^468^ Analytical approaches currently used for MRV can be broadly categorized into two classes. Flux-based methods quantify CDR through observed changes in air–sea CO_2_ exchange, while fate-based methods track the persistence of macroalgal-derived carbon in POC, RDOC, or bicarbonate forms. Complementary life cycle assessments are needed to determine whether net climate benefits are achieved once upstream and downstream emissions are included. Emerging international pilot programs are now providing testbeds for MRV system design, establishing practical standards for verification and cross-comparison among diverse CDR approaches.

#### 5.4.1 Quantification of Ecosystem Flux-Based CO_2_ Removal

An ecologically grounded approach to quantifying macroalgae-based CDR involves direct measurement of net CO_2_ fluxes across the air–sea interface at high temporal and spatial resolution. The total net CO_2_ uptake is commonly expressed as net ecosystem production or net community production, depending on measurement scale and methodological frameworks.^466,469–472^ These flux-based metrics integrate both photosynthetic carbon uptake and community respiration, providing a comprehensive measure of the net carbon balance with macroalgal habitats.

During growth, macroalgal assimilation of aqueous CO_2_ and HCO_3_^–^ enhances the air–sea CO_2_ gradient, stimulating atmospheric CO_2_ uptake. Conversely, senescence and subsequent decomposition release CO_2_, potentially driving local outgassing. When CO_2_-depleted waters formed during growth are advected away, they may continue to absorb atmospheric CO_2_ until equilibrium is restored, whereas rapid subduction of these waters can neutralize CO_2_-rich subsurface layers. Both processes influence large-scale ocean-atmosphere CO_2_ exchange, though their contribution to durable atmospheric CO_2_ removal remains debated.^466^

Flux-based CDR assessments employ in situ measurements of *p*CO_2_ and carbonate chemistry to monitor CO_2_ exchange over extended temporal and regional scales. High-resolution O_2_ flux data derived from aquatic eddy covariance techniques^473–476^ can also be converted to DIC flux using diel O_2_–DIC relationships.^477,478^ Localized incubation experiments enclosing monospecific macroalgal stands are also used to quantify integrated metabolic activity, including microbial and invertebrate respiration.^474,477,478^ Scaling efforts increasingly rely on complementary hyperspectral or RGB imagery acquired from satellites, drones, and autonomous platforms.^468,478,479^ However, extrapolating from patch-scale measurements to heterogeneous coastal mosaics must be approached with caution, as strong spatial and temporal variability, especially combined with nutrient reallocation, complicates flux attribution.

#### 5.4.2 Quantification of Fate-Based CO_2_ Storage

Whereas flux-based methods assess net ecosystem carbon balance, fate-based approaches quantify the proportion of carbon fixed by macroalgae that ultimately contributes to long-term storage. These approaches provide species- and habitat-specific insights into macroalgae-based CDR mechanisms but remain limited by sparse ecological and geochemical data. NPP or areal biomass serve as scalable proxies for the initial carbon fixation. NPP is typically derived from photosynthesis–irradiance (P–E) relationships integrated with light availability,^477,480,481^ while areal biomass estimates rely on diver surveys and underwater imaging. However, neither metric alone reflects storage, as they do not capture the persistence of fixed carbon. Quantifying the conversion efficiency from NPP to POC export and burial, RDOC production, and HCO_3_^–^ generation is therefore essential for fate-based CDR assessments.

Species-specific traits strongly influence these partitioning pathways. POC burial is commonly estimated using methods adapted from BCEs—including sediment organic carbon content, age dating, and accumulation rate analyses,^482^—while RDOC yield is measured as the fraction of DOC resistant to microbial degradation over extended time scales (e.g., > 200 days).^410,413^ HCO_3_^–^ formation, though seldom quantified, can be inferred from carbonate system variability after correcting for nutrient and organic contributions.^382,383^

Among these pathways, POC export and burial are generally regarded as the dominant mechanisms of long-term carbon storage in macroalgal systems.^483^ Yet their quantification remains difficult: rocky substrates inhibit local sediment accumulation, and exported POC is often advected far from its source.^392^ Tracking this exported carbon—identifying its origin, transformation, and depositional environment—is crucial but remains uncertain.^379^ Even when bulk losses are estimated (e.g., ∼ 45% of NPP in cultivated kelp released as DOC and POC combined),^484^ only a fraction of this carbon persists. Determining the ultimate fate of exported POC remains a central challenge, as its CDR potential depends on both particle-specific properties (e.g., density, sinking velocity, degradability, composition) and environmental factors such as water depth, hydrodynamics, turbulence, and meteorological forcing.^485^

Emerging analytical tools—including environmental DNA, stable isotope tracers, species-specific biomarkers, and molecular fingerprinting—offer promising means to trace the sources and fate of macroalgae-derived organic carbon.^486^ However, methodological inconsistencies—such as expressing POC burial per unit area versus RDOC per unit biomass—impede integration into unified CDR estimates. Combined with strong habitat heterogeneity and physical transport constraints, these challenges emphasize the need for standardized, scalable monitoring frameworks to enable robust fate-based verification of macroalgal CDR.

#### 5.4.3 Global Measurement, Reporting, and Verification Practices

Macroalgae-based CDR is not yet formally incorporated into global carbon accounting frameworks, but emerging pilot initiatives are beginning to establish methodologies for MRV. In Japan, for example, a blue carbon offset crediting scheme has been developed.^487^ Within this framework, additional storage from macroalgal forest restoration is estimated using removal coefficients, whereas aquaculture-based CDR is quantified more directly as NPP multiplied by the fraction of residual carbon retained within the ocean reservoir.^487,488^

In parallel, the European Union’s proposed QU.A.L.ITY criteria^467^—requiring carbon removal to be quantifiable, additional, durable, and sustainable—offer a conceptual basis for incorporating macroalgal CDR into voluntary carbon markets. Lessons can also be drawn from coastal wetland restoration programs. Australia, for instance, recognizes coastal wetlands as BCEs under its Emissions Reduction Fund, where the Blue Carbon Accounting Model is applied to compare baseline and postrestoration conditions and to evaluate the durability of stored carbon.^489^ Although macroalgal systems differ fundamentally from coastal wetlands in their growth—harvest dynamics and potential for nutrient redistribution, these precedents illustrate that existing MRV frameworks can be adapted to accommodate macroalgae-based CDR. Building upon established principles of quantifiability, additionality, and permanence, future accounting methodologies must be tailored to the distinct biological, geochemical, and physical processes governing macroalgal carbon storage.

#### 5.4.4 Life Cycle Assessment

As with other CDR approaches, the net effectiveness of macroalgae-based CDR must be assessed by balancing gross CDR against all associated carbon emissions (eq 45 in Section 4.4.3). Emissions arise from aquaculture infrastructure and operations—including the production and maintenance of ropes, buoys, and other materials, waste management, electricity use, nutrient supplementation, and vessel fuel for deployment and harvesting^490^—as well as from biogeochemical feedbacks such as enhanced calcification, suppressed phytoplankton production, and the release of greenhouse gases including CH_4_ and N_2_O.^424,452,460,461^

Life cycle assessments (LCAs) indicate that kelp aquaculture can operate with relatively low direct emissions; values on the order of ∼ 57.5 kg CO_2_-eq per ton of fresh biomass have been reported, particularly when infrastructure components are recycled.^490^ However, because one ton of macroalgal biomass contains only ∼ 121 kg CO_2_ (assuming 11% dry weight and 30% carbon content), even deliberate deep-ocean sinking would store limited amounts of carbon relative to total emissions.

Most existing LCAs emphasize product-based utilization pathways—such as biofuels, bioplastics, biochar, or animal feed—but these remain constrained by high processing emissions and limited cost-effectiveness under current technologies.^387–391^ Comprehensive, system-level LCAs are therefore required to rigorously assess whether macroalgae-based CDR can achieve verifiable, durable, and scalable climate benefits once all operational, biochemical, and downstream fluxes are considered.

## 6 Concluding Remarks

This review has examined the marine carbonate system both as a natural regulator of anthropogenic CO_2_ and as the chemical foundation for engineered mCDR strategies. By integrating discussions of carbonate chemistry and its analytical basis (Section 2), the dynamics and variability of the natural oceanic CO_2_ sink (Section 3), and two representative mCDR approaches—OAE (Section 4) and macroalgae-based strategies (Section 5)—we demonstrate how the analytical carbonate framework provides a unifying lens for understanding natural regulation, anthropogenic perturbation, and engineered interventions in the marine carbon cycle.

OAE represents a chemically grounded approach that elevates seawater alkalinity via the dissolution of alkaline materials, thereby lowering surface *p*CO_2_ and enhancing oceanic CO_2_ uptake. Its scalability, however, is constrained by challenges related to dissolution kinetics, trace-metal composition, and secondary mineral reactions. Macroalgae-based CDR, in contrast, relies on biological CO_2_ fixation and subsequent storage through multiple pathways, including POC export and burial, RDOC storage, and bicarbonate production. Despite growing experimental interest, the persistence, quantification, and feedback associated with these processes remain uncertain. Both strategies demand rigorous MRV protocols, supported by comprehensive LCA to ensure additionality, durability, and scalability. Predictive models linking air–sea CO_2_ exchange, ocean circulation, and carbon storage must be validated through coordinated laboratory and field programs, accompanied by systematic evaluations of environmental impacts, societal acceptance, and regulatory approval. Although recent pilot studies and advances in MRV mark meaningful progress, no current mCDR strategy has yet demonstrated a durable net-negative carbon balance.

These interventions also challenge the analytical foundations of the carbonate chemistry framework itself. Sample pretreatment may fail to preserve in situ conditions: elevated alkalinity can induce secondary reactions during handling, while high organic loads can bias TA measurements. Moreover, engineered perturbations can push pH, TA, and DIC beyond the calibration ranges of standard analytical methods, complicating accurate characterization of deployment site. Shifts in elemental ratios, ionic strength, and acid–base equilibria—arising from OAE-derived ion inputs or macroalgal nutrients and organic release—further complicate carbonate speciation analyses and thermodynamic modeling. Accurate assessment of CDR efficacy will require parallel advances in analytical protocols and modeling frameworks that underpin marine carbon cycle research.

The ocean remains Earth’s largest and most dynamic carbon sink, absorbing a substantial portion of anthropogenic CO_2_ and thereby delaying climate change. It is thus a natural focus for CDR innovation. However, the central challenge lies in translating its intrinsic biogeochemical mechanisms—mineral weathering, alkalinity generation, photosynthetic fixation, and carbon export—into scalable, durable interventions that preserve ecosystem integrity. Viewing the ocean not only as a carbon sink but also as a source of mechanistic insight will enable the design of engineered solutions that operate in harmony with natural processes, advancing the broader goal of long-term climate stabilization.

## Abbreviations

C^ANT^: anthropogenic CO_2_
HCO_3_^–^: bicarbonate ion
BCE: blue carbon ecosystem
CaCO_3_: calcium carbonate
Ca­(OH)_2_: calcium hydroxide
CaO: calcium oxide
CO_2_: carbon dioxide
CDR: carbon dioxide removal
CO_3_^2–^: carbonate ion
H_2_CO_3_: carbonic acid
DBL: diffusive boundary layer
DIC: dissolved inorganic carbon
DOC: dissolved organic carbon
DOM: dissolved organic matter
O_2_: dissolved oxygen
η_CO2_: efficiency factor
eMLR: extended multiple linear regression
*f*CO_2_: fugacity of CO_2_
mCDR: marine carbon dioxide removal
Mg­(OH)_2_: magnesium hydroxide
MRV: monitoring, reporting, and verification
NPP: net primary production
OAE: ocean alkalinity enhancement
*p*CO_2_: partial pressure of CO_2_
POC: particulate organic carbon
*P*: pressure
*S*: salinity
Ω: saturation state
RDOC: refractory DOC
TA: total alkalinity
*T*: temperature
